# Remains of Leatherback turtles, *Dermochelys coriacea*, at Mid-Late Holocene archaeological sites in coastal Oman: clues of past worlds

**DOI:** 10.7717/peerj.6123

**Published:** 2018-12-17

**Authors:** John G. Frazier, Valentina Azzarà, Olivia Munoz, Lapo Gianni Marcucci, Emilie Badel, Francesco Genchi, Maurizio Cattani, Maurizio Tosi, Massimo Delfino

**Affiliations:** 1 National Museum of Natural History, Smithsonian Institution, Department of Vertebrate Zoology–Amphibians & Reptiles, Washington, D.C., USA; 2 Faculty of Archaeology, Leiden University, Leiden, Netherlands; 3 UMR 7041 Archéologie et Sciences de l’Antiquité, Equipe “du Village à l’Etat au Proche et Moyen Orient”, Maison de l’Archéologie et de l’Ethnologie, Nanterre, France; 4 Department of History and Cultures, University of Bologna, Bologna, Italy; 5 Dipartimento di Scienze della Terra, Università di Torino, Torino, Italy; 6 Institut Català de Paleontologia Miquel Crusafont, Universitat Autónoma de Barcelona, Cerdanyola del Valles, Barcelona, Spain

**Keywords:** Bronze Age, Ra’s al-Hamra, Neolithic, Zooarcheology, Ossicles, Taphonomy, Marine turtles, Ra’s al-Hadd

## Abstract

Small, irregular isolated bones identified as remains of leatherback turtles (*Dermochelys coriacea*) were recovered from Mid to Late Holocene sites at Ra’s al-Hamra and Ra’s al-Hadd, coastal Oman. These provide the third instance of this animal being documented from any prehistoric site anywhere, and the records provide one of the oldest, if not the oldest, dates for this distinctive chelonian—even though they do not refer to fossils. Decades of research in this region has yielded vast amounts of archeological information, including abundant evidence of intense exploitation and utilization of marine turtles from about 6,500 to 4,000 BP. During part of this period, turtle remains in human burials have been extraordinary; the turtle involved, *Chelonia mydas*, has been abundant in the region during modern times. Yet despite intense and varied forms of prehistoric marine resource exploitation, and major, long-term archeological work, no other turtle species has been previously authenticated from these, or other coastal sites. The documentation of remains of the largest and most distinctive of living marine turtles, *D. coriacea*, at Ra’s al-Hamra and Ra’s al-Hadd, presented herein, provide detailed information that serves as the basis for future interpretations and discussions regarding incomplete, disarticulated remains from the Mid to Late Holocene, particularly in reference to taphonomic questions and diverse environmental conditions.

## Introduction

*Dermochelys coriacea* (Vandelli, 1761) is the largest living turtle, and the only extant member of the family Dermochelyidae, presently considered to include nine genera with a total of 17 species, the oldest of which dates back to at least the Early/Lower Campanian (Late Cretaceous), nearly 84 Ma (Supplemental Information 1). At most, the fossil record of *D. coriacea* may include one, or two, small isolated bones, from the Plio-Pleistocene Lee Creek Mine, North Carolina, USA. These irregular ossicles (alternately called “dermal ossicles,” “osteoderms,” or “platelets”) were reported in an unpublished thesis (Köhler, 1996) which was briefly mentioned in two recent paleontological publications (Karl, Gröning & Brauckmann, 2012a: 166; Karl, Lindow & Tütken, 2012b: 214), and only tentatively confirmed recently (Supplemental Information 2). However, consistently *D. coriacea* is regarded to have no fossil record (Wood et al., 1996, 2009; F. de Lapparent de Broin *in litt.* to JF August 22, 2017; J. Parham *in litt.* to JF September 20, 2017; R. Hirayama *in litt.* to JF September 11, 2018; R. Weems *in litt.* to JF September 12, 2018).

Similarly, despite its circumglobal distribution, distinctive morphology, and large body size, growing to over 1.5 m in length and up to a tonne in body weight (Frazier, Gramentz & Fritz, 2005: 249–251, 271 ff; Eckert et al., 2012: 6–8, 53), this animal has been reported only twice from prehistoric archeological sites, both later than 200 AD and from the Caribbean (Frazier, 2005). The fact that hundreds of coastal sites, around the world, have been studied by archeologists and zooarcheologists emphasizes the rarity of these archeological specimens.

**Figure 1:**
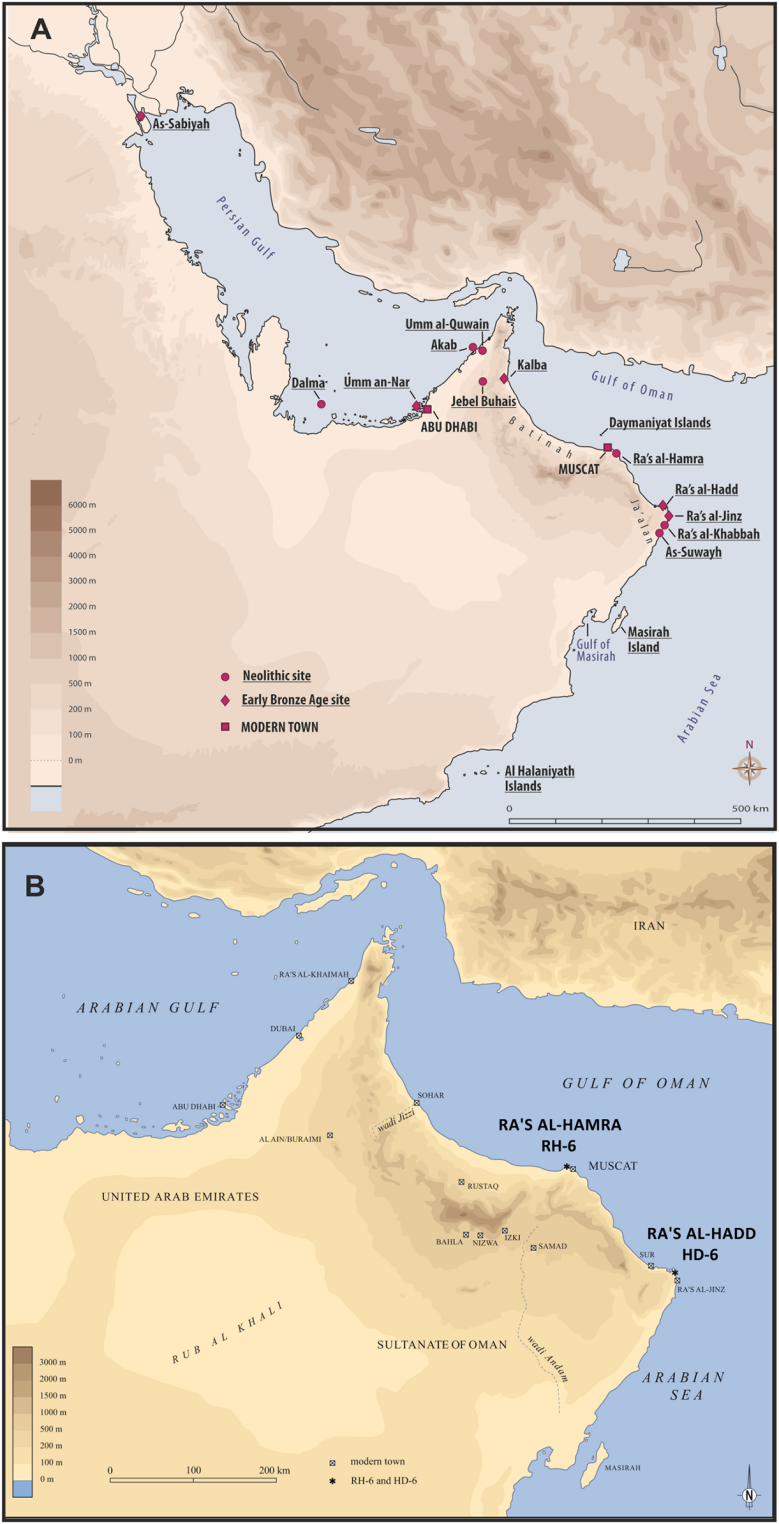
Two coastal headlands are central to the present study: Ra’s al-Hamra, on the eastern limit of the sandy coast of Batinah, and Ra’s al-Hadd, at the easternmost extreme of the Ja’alan region. (A) The localities mentioned in the text, mainly the archeological sites: As-Sabiyah (Kuwait), Dalma, Umm an-Nar, Akab, Umm al-Qawain, Jebel al-Buhais, and Kalba (United Arab Emirates); Ra’s al-Hamra, on the Batinah coast, Ra’s al-Hadd, Ra’s al-Jinz, Ra’s al-Khabbah, and As-Suwayh, all on the coast of Ja’alan (Oman), as well as the offshore Daymaniyat Islands, Masirah Island, and Al Halaniyath Islands of Oman; Neolithic and Early Bronze Age sites are distinguished (figure by V. Azzarà, based on a base map of H. David). (B) The most easterly extension of the Arabian Peninsula, and Oman, showing the geographic relationship between Ra’s al-Hamra, with RH-6, and Ra’s al-Hadd, with HD-6 (figure by L.G. Marcucci, based on a base map of H. David).

The Sultanate of Oman, in the south-eastern corner of the Arabian Peninsula, hosts hundreds of prehistoric sites, some estimated to be more than 10,000 years old (Charpentier, 2008; Charpentier & Crassard, 2013, Charpentier et al., 2016; Rose et al., 2018); Paleolithic, Neolithic, Bronze Age, Iron Age, and Islamic sites are well represented (Uerpmann & Uerpmann, 2003; Cleuziou & Tosi, 2007). Lagoonal and marine environments are regarded to have been major sources of food and materials for all prehistoric coastal populations—even after the development of agricultural and pastoral economies, and these resources continue to be important today (El Mahi, 2000; Uerpmann & Uerpmann, 2003; Cleuziou & Tosi, 2007; Azzarà, 2012; Berger et al., 2013; Zazzo, Munoz & Saliège, 2014; Munoz, 2017). Two coastal headlands are central to the present study: Ra’s al-Hamra, on the eastern limit of the sandy coast of Batinah, and Ra’s al-Hadd, at the easternmost extreme of the Ja’alan region (Fig. 1).

**Figure 2:**
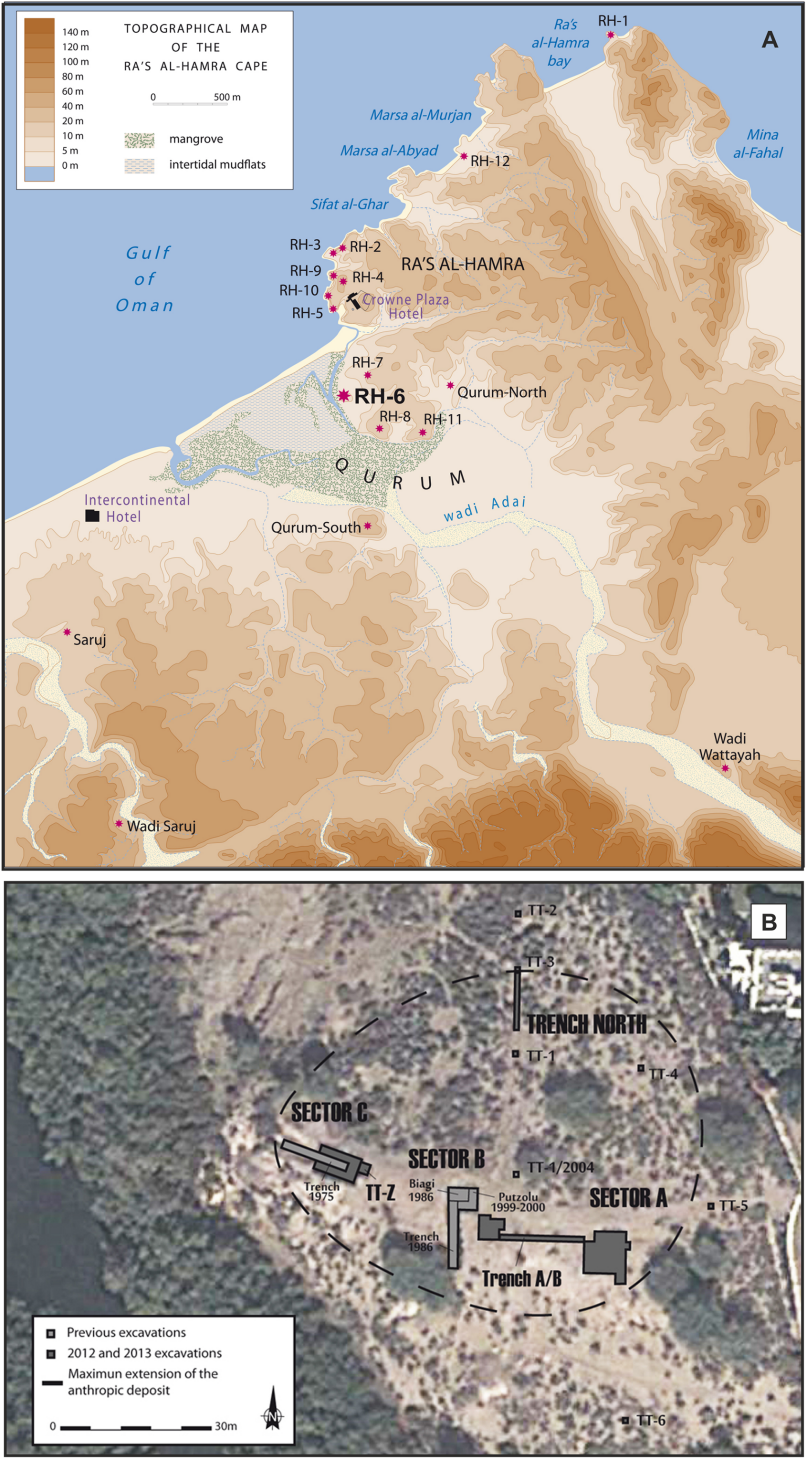
The archeological sites and sandy beaches at Ra’s al-Hamra. (A) The Ra’s al-Hamra and Qurum area, showing locations of archeological sites mentioned in the text, as well as present-day sand beaches in the area: seaward and west of RH-6, Sifat al-Ghar, Marsa al-Abyad, Marsa al-Murjan, Ra’s al-Hamra bay, and Mina al-Fahal (figure by L.G. Marcucci, based on a base map of H. David; note: RH-9 is not an ancient site, see Supplemental Information 3). (B) General excavation plan of Ra’s al-Hamra 6, showing Sector A, Sector B, Sector C, TT-Z, and Trench North (modified by L.G. Marucci; Google, 2013 DigitalGlobe).

Archeological research at Neolithic sites along coastal Oman began in 1973 at Ra’s al-Hamra, with the first excavations in 1977 (Tosi, 1989; Marcucci, 2014); research on this limestone platform has continued to the present (Durante & Tosi, 1977; Salvatori, Coppa & Cucina, 2007; Marcucci et al., 2011, 2014; Zazzo et al., 2016). This headland is within the urban area of Muscat, the nation’s capital. A total of 12 different archeological sites have been enumerated at Ra’s al-Hamra (Fig. 2; Supplemental Information 3), but primarily because of coastal development, nine sites were destroyed before systematic work by trained archeologists could begin (Durante & Tosi, 1977: 141–142; Salvatori, 1996; Uerpmann & Uerpmann, 2003; Munoz, 2014). Radiocarbon dating from Ra’s al-Hamra sites indicates a maximum span from 5608 BC to 55 AD (Biagi, 1994; Uerpmann & Uerpmann, 2003; Zazzo, Munoz & Saliège, 2014; Zazzo et al., 2016; Munoz, 2014), although the shell middens and the period of intense habitation are estimated to date between 6,000 and 3,000 BC (Cleuziou & Tosi, 2007; Munoz, 2014: 134, fig. A4).

Voluminous evidence is available on Neolithic subsistence, settlement, and funerary practices; skeletal remains of marine animals have been abundantly documented; the dominance of tuna bones in middens is notable (Uerpmann & Uerpmann, 2003, 2007; Uerpmann, 2003). Site RH-5, estimated to have been occupied from before 4,000 until about 3,000 BC, includes a large human cemetery, where at least 149 graves have been excavated (Salvatori, 2007; Salvatori, Coppa & Cucina, 2007). Recent microstratigraphic excavations in RH-5 graves yielded repeated evidence of nets, some evidently of large size (Munoz, 2014: 169). Although there is no physical evidence of boats or other types of vessel, evidence of the “material culture”—particularly artifacts interpreted to have been fishing implements—and zooarcheological evidence—including remains of large pelagic fishes as well as sharks—have prompted trained archeologists to propose that some kind of watercraft was used at RH-5 (Uerpmann & Uerpmann, 2003; Uerpmann, 2003; Salvatori, Coppa & Cucina, 2007; Marcucci et al., 2011). Some of the most remarkable grave goods at RH-5 are skeletal remains of green turtles, *Chelonia mydas* (Linnæus, 1758), whose bones have been recovered from 80 graves. Turtle crania, or parts of crania, are remarkably common, appearing in nearly 13% of excavated graves: as many as 29 skulls have been recovered from a single grave, and as many as 34 lower jaws were found in another grave: hence, the conclusion that *Chelonia mydas* was an important resource for nutritional as well as cultural needs (Uerpmann & Uerpmann, 2003; Salvatori, 2007; Salvatori, Coppa & Cucina, 2007; Munoz, 2014). Although marine turtle remains are well documented from human graves around the world, their predominance in RH-5 burials is unique (Frazier, 2003, 2005); and some authors have called the RH-5 people “seasonal turtle worshipers” (Tosi, 1989: 152; Cleuziou & Tosi, 2007: 76, fig. 58 ff).

The older RH-6 site at Ra’s al-Hamra is nowadays located on the eastern edge of a mangrove within the Qurum Nature Reserve, presently about 600 m inland from the sea, and about 700 m south of RH-5 (Fig. 2). RH-6 is dominated by a massive midden, nearly two m high in some places, associated with settlement areas and a graveyard. This midden was first identified in 1975 (Tosi, 1975; Durante & Tosi, 1977), and then more thoroughly investigated during several excavation seasons in the 1980s and late 1990s and early 2000s (Biagi, 1999; Marcucci, 2015). Most recently, RH-6 was studied in 2012 and 2013 in response to pending construction of an archeological park planned at Ra’s al-Hamra (Marcucci, 2017: 6). Several studies (Marcucci et al., 2014: fig. 2; Marcucci, 2015: 350–353) have concluded that the thick, well-packed layers of marine shells and fish bones that constitute the RH-6 midden are the result of intense prehistoric, year-round exploitation of lagoon and marine animals; the evidence supporting this proposition includes: remains interpreted as ancient features (e.g., post holes, hearths, refuse pits) and human burials, as well as hard objects, interpreted to have been made or modified by human agency (“artifacts”) (e.g., widely found net sinkers, fish hooks, and gorges), and also remains of living organisms, interpreted to have been impacted in diverse ways by human agency (“ecofacts”) (e.g., numerous hearths with concentrated remains of molluscs and fishes—signs of food preparation) As in the case of RH-5, there are abundant remains of fish bones, with trevallies (Carangidae) and tunas predominating; hence, archeologists have argued that some kind of watercraft was used at RH-6 (Uerpmann & Uerpmann, 2003; 198–199; Marcucci, 2015: 390, 391, 450 fn 1361). Based on radiocarbon dating, the RH-6 settlement is estimated to have been occupied from about 5,603 to 4,473, 2σ cal BC (Zazzo et al., 2016); and it is one of the earliest known human occupations, both for Ra’s al-Hamra and coastal Oman. However, the radiocarbon dates also indicate that the RH-6 graveyard was in use later, after a 500- to 700-year hiatus, around 3,996–3,830, 2σ cal BC (Zazzo et al., 2016).

“Interestingly, turtles and sea mammals were rare or nonexistent at RH-6 as compared to the later sites” [viz RH-4, RH-5, and RH-10] (Uerpmann & Uerpmann, 2003: 247). The only published information on turtle remains recovered from RH-6 seems to be an enigmatic comment in Biagi (1999: 66, fig. 19–44) that reported and illustrated “a turtle bone with a double, diverging perforation,” and a brief comment in Marcucci et al. (2014: 245) that “a few turtle bones were found inside” Pit 8 (of Sector C, attributed to Period II).^1^1From what can be seen in a photo of a wall of Pit 8 (Marcucci, 2015: fig. 6), one of these turtle bones is at least 13 cm long and up to one cm thick, and appears to be from a “hard-shelled” (cheloniid) turtle (J. G. Frazier, 2018, personal observation). In addition, Marcucci’s unpublished PhD dissertation (2015: 392–398) gave a brief, preliminary description of marine turtle remains at RH-6, including *D. coriacea* ossicles reported in detail in the present article.

**Figure 3:**
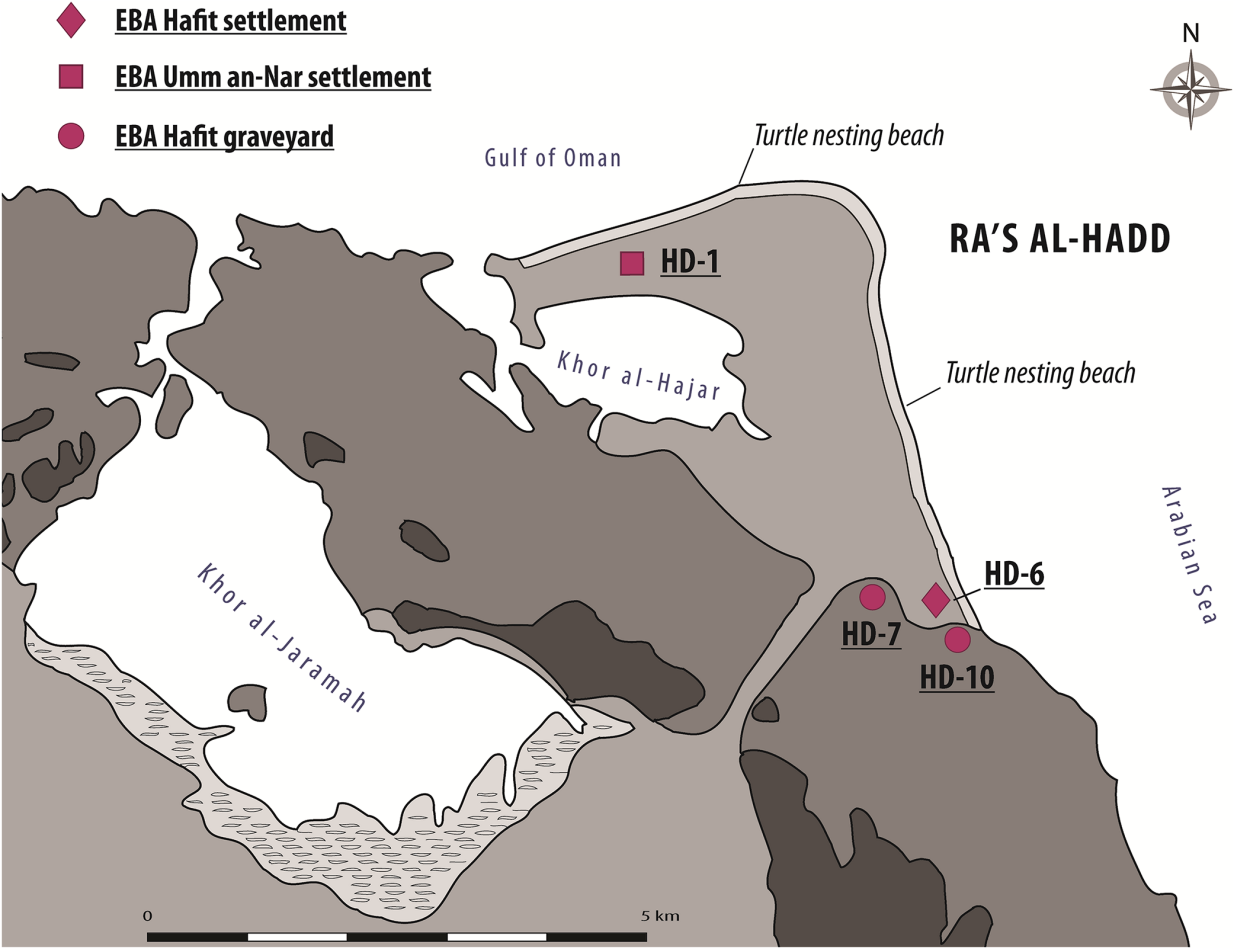
The Ra’s al-Hadd area, showing locations of archeological sites mentioned in the text, as well as present-day turtle nesting beaches. EBA, Early Bronze Age; Hafit Period refers to about 3,200 to 2,600 BC; Umm an-Nar Period refers to about 2,600 to 2,000 BC (figure by V. Azzarà).

Ra’s al-Hadd, the second area of importance for the present study, lies about 300 km SE of Ra’s al-Hamra; it is the eastern-most extension of Oman and the Arabian Peninsula, forming the limit between the Gulf of Oman to the north and the Arabian Sea to the south (Figs. 1 and 3). Archeological research began in 1987, with increased activity after 1995 (Azzarà, 2015: 113–114). More than 13 archeological sites have been identified at Ra’s al-Hadd, which range from Neolithic to Islamic; nearly all of these are regarded to be younger than the Ra’s al-Hamra sites.

Although at least three different sites at Ra’s al-Hadd are interpreted to have been occupied after about 2,500 BC; site HD-6, central to this study, is the only settlement thought to have been occupied earlier (∼3,100–2,700/2,600 BC). HD-6 stands on a sand bar at the edge of a paleo-lagoon, about 200 m from the Arabian Sea and about 300 m south of the present-day village of Ra’s al-Hadd. HD-6 was excavated from 1996 to 2015 (Tosi & Cattani, 1997; Cattani & Cavulli, 2004; Azzarà, 2009, 2012, 2013). The first occupation (Phase I-1), was followed by three subsequent phases (I-2, I-3, and I-4), each with adobe and stone structures, and further divided into several subphases (i.e., I-2a to I-2e, I-3a to I-3e, and I-4a to I-4b) based on stratigraphic-structural sequences (Azzarà, 2013, 2015). The timings of these phases/subphases is supported by recent C^14^ analysis of charcoal from the first to nearly the last occupational phase which gave radiocarbon estimates from 3,090–2,870 to 2,620–2,330, 2σ cal BC (Azzarà, 2012: fn 3, 2013: fn 1, fig. 3, 2015: 126–133).

**Figure 4:**
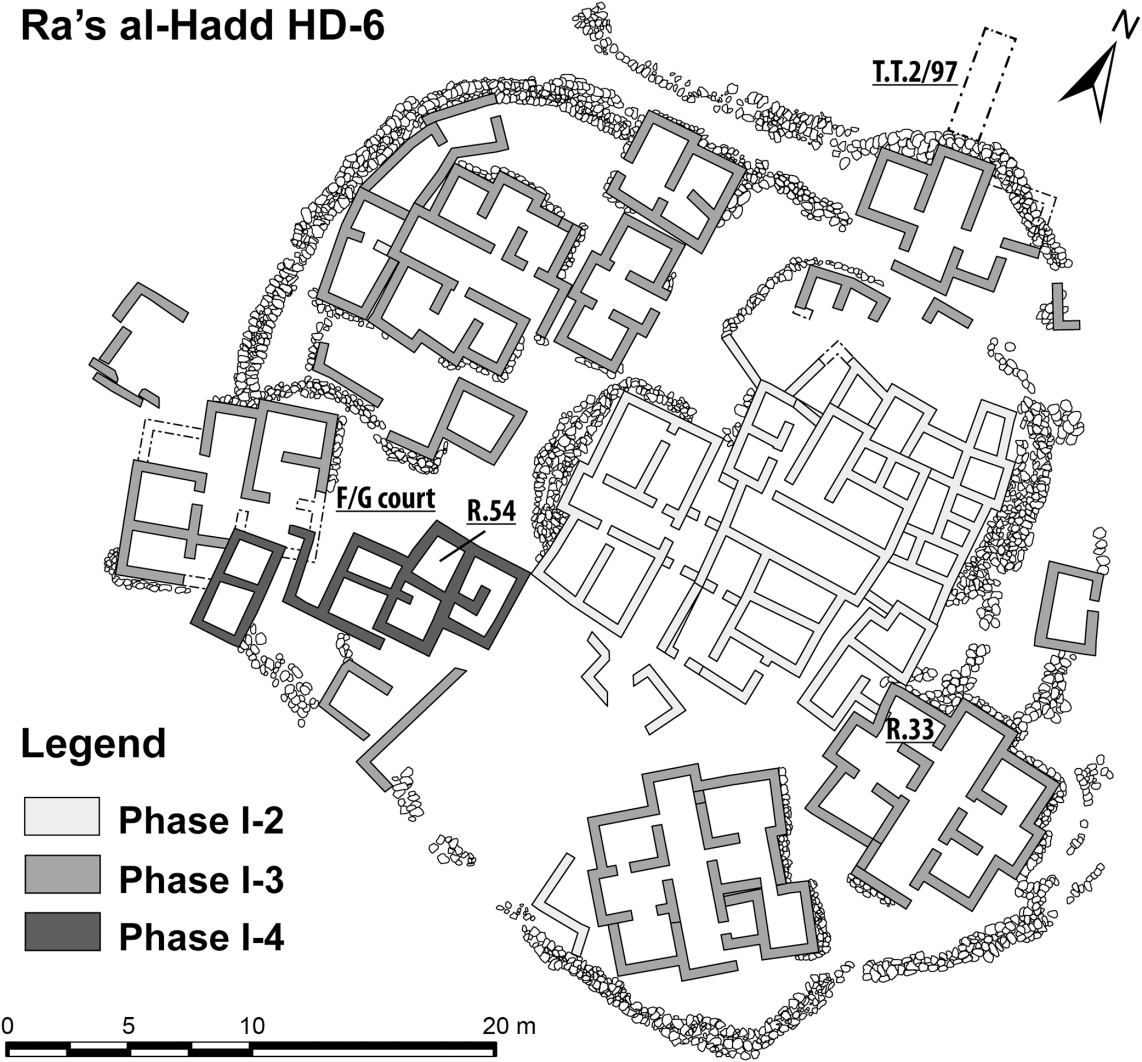
Plan of the Early Bronze Age occupation at Ra’s al-Hadd HD-6, Oman. Locations of key areas mentioned in the text: Room 33, Room 54, Courtyard F/G, and T.T.2/97 (figure based on Azzarà, 2015).

The settlement at HD-6 was larger and more complex than those at Ra’s al-Hamra. Remains of buildings (Fig. 4), with apparent workshops and storage facilities, indicate activities such as production of domestic supplies and jewelry, mainly stone- and shell-beads and rings (Azzarà, 2009, 2012; Hilbert & Azzarà, 2012: 18–24). Abundant faunal evidence indicates that intense fishing occurred during all occupational phases at HD-6, and included a wide variety of animals, invertebrates and vertebrates, pelagic and benthic. Artifacts interpreted as net sinkers, vestiges of large nets, and fish hooks are common; and abundant evidence supports the interpretation of prehistoric preparation of marine animals, particularly fishes and marine turtles. The material culture as well as the faunal remains (e.g., sharks, tuna, and other large pelagic fishes) indicate that some sort of watercraft was used (Tosi et al., 2002; Cleuziou & Tosi, 2007: 58; Azzarà, 2012; 255). Oil from dolphins (Delphinidae) and marine turtles (evidently *Chelonia mydas*) is thought to have been extracted in large quantities for both storage and trade (Mosseri-Marlio, 1998a, 1998b, 2000, 2002). During modern times the beach seaward of Ra’s al-Hadd has been of major importance for nesting *Chelonia mydas*, with up to 10,000 females estimated to nest each year (Salm & Salm, 1991; Gasperetti et al., 1993; Salm, Jensen & Papastavrou, 1993).

HD-6 has yielded numerous signs of trade. The scale of production of shell- and stone-beads indicates that they were produced, at least partially, for trade, evidently to obtain different goods from other communities, a hypothesis supported by copper objects (including fishing hooks) recovered from HD-6, made from copper that apparently came from the interior of the country. Notably, later sites at Ra’s al-Hadd show clear material evidence of trade with overseas regions, such as Mesopotamia and the Indus Valley; indeed, Ra’s al-Hadd has been called the “main [prehistoric] port for international trade” (Cleuziou & Tosi, 2007: 235).

HD-6 is the most complex coastal settlement from Oman known from before 2,500 BC (Azzarà, 2013). Within one km of HD-6 there are numerous “cairn,” or “turret,” graves, from the same period, located in sepulchral areas, including sites HD-10 (Salvatori, 2001), and HD-7, where fish remains were recovered from all tombs that have been studied; although other animal remains (including turtle bones) have been found, it is unclear if these were part of the burial ritual, or simply included with infill (Munoz, 2014: 231–232).

During modern times the coasts and waters of Oman have hosted all five of the circumglobal species of marine turtles, and the numbers of three of the species that nest in Oman are globally important. Most abundant, and widely distributed, is the green turtle, *Chelonia mydas*, which is recorded to nest on at least 275 beaches along approximately 2,000 km of coast. An estimated 90% of nesting occurs along the Arabian Sea; and the 45 km stretch of beach from Ra’s al-Hadd south to Ra’s al-Khabbah (Fig. 1) is of particular importance. There are an estimated 20,000 nesters per year in Oman, of which half nest in the Ra’s al-Hadd area; nesting is year round in Oman. During present times there has been little nesting on the Batinah coast, but this may be a result of modern-day human disturbances. The mainland coasts of southern Oman provide important feeding areas for *Chelonia mydas* (Salm & Salm, 1991; Gasperetti et al., 1993; Salm, Jensen & Papastavrou, 1993; Baldwin & Al Kiyumi, 1999).

The island of Masirah, off the south-eastern coast of Oman (Fig. 1), hosts one of the largest nesting populations in the world of loggerhead sea turtles, *Caretta caretta* (Linnæus, 1758). They also nest on other islands, primarily the Al Halaniyat Islands, and on nearly 200 mainland beaches of the Arabian Sea, particularly in the south-western extreme of Oman. The southwest of the country also seems to provide important feeding areas. There are occasional records of *Caretta caretta* from the Muscat area (Ross & Barwani, 1982; Salm & Salm, 1991; Salm, Jensen & Papastavrou, 1993; Baldwin, Hughes & Prince, 2003; Conant et al., 2009).

While the numbers are nowhere near as large as for *Chelonia mydas* or *Caretta caretta*, there are abundant records of the hawksbill, *Eretmochelys imbricata* (Linnæus, 1766) nesting in Oman, especially on the Daymaniyat islands (in the Muscat area, Fig. 1), and less so on Masirah Island. With nearly 1,000 nesters per year, the number of *E. imbricata* nesting in Oman is of regional and global significance. Known feeding areas of importance for this turtle are around Masirah Island and in the far west of Oman (Salm & Salm, 1991; Gasperetti et al., 1993; Salm, Jensen & Papastavrou, 1993; Rees & Baker, 2006; Pilcher et al., 2014).

The fourth species known to nest in Oman is the olive ridley, *Lepidochelys olivacea* (Eschscholtz, 1829). There is scattered nesting on the mainland coast of the Arabian sea, but most known nesting, estimated to be more than 100 females per year, is on Masirah Island. The waters around this island seem to be important feeding areas; and there are accounts of unexplained large die-offs of these turtles that have stranded along the coasts of the Gulf of Masirah (Salm & Salm, 1991; Gasperetti et al., 1993; Salm, Jensen & Papastavrou, 1993; Rees & Baker, 2006; Rees et al., 2012).

The leatherback, *D. coriacea*, occurs in Oman, but there are no accounts, contemporary or historical, of nesting. There may be some feeding in Omani waters, particularly in the western extreme. There are occasional reports of these turtles washing up dead on the Arabian Sea coast of the country. Oman is not considered to be an important area for this species, and some authors regard this turtle as a vagrant in Oman (Salm & Salm, 1991; Gasperetti et al., 1993; Salm, Jensen & Papastavrou, 1993; Baldwin & Al Kiyumi, 1999; Hamann et al., 2006).

During modern times local fishermen have caught marine turtles on beaches and in the sea with harpoons, gaffs, and nets; the fat is reputed to be a preferred part. At least four species of turtles are known to have been hunted during recent times, but *Chelonia mydas* comprises more than 95% of the catch. Eggs are also consumed (Salm, Jensen & Papastavrou, 1993; Baldwin & Al Kiyumi, 1999). To date, only *Chelonia mydas* has been documented from archeological sites in Oman, including RH-6 and HD-6 (Frazier, 2003, 2005; Uerpmann & Uerpmann, 2003).

## Materials and Methods

The zooarcheological specimens discussed herein derive from two localities of coastal Oman: Ra’s al-Hamra and Ra’s al-Hadd (Fig. 1); work at sites RH-6 and HD-6, has been carried out under two different frameworks. Although the research objectives and conditions at the two sites have not been identical, investigations at both sites have employed similar sampling and excavation procedures, such as spatial matrix sampling and stratigraphic sequencing as described by Harris (1997). These widely recognized standard protocols in the field of archeology have thus providing common ground for data extrapolation and comparison between the two Omani sites. The zooarcheological specimens described in this paper were compared with known reference specimens of both recent *D. coriacea* and fossil *Psephophorus polygonus* Meyer, 1847. The RH-6 and HD-6 specimens were examined macroscopically; no detailed microscopic analyses were undertaken.

### Ra’s al-Hamra

Although the RH-6 midden was first identified in 1975, with subsequent excavations over nearly four decades, the specimens reported herein were only retrieved during the 2012 and 2013 seasons; four loci were investigated: Sectors A, B, and C, as well as Trench North (Fig. 2A). All but the last-named were worked during earlier excavations (Marcucci et al., 2014; Marcucci, 2015). Extensive excavation of Sector A, at the eastern edge of the anthropogenic deposit, revealed two occupational periods, the first of which is the earliest known period of occupation at RH-6 (Marcucci et al., 2014; Zazzo et al., 2016). At Sector B (a graveyard area), which is toward the center and top of the mound, only the upper-most layers of the stratigraphic sequence were investigated. Six funerary structures, part of a larger cluster of graves, were excavated. Sector C, near the western extreme of the site, showed six periods of occupation, the longest occupational sequence for RH-6. Test trench TT-Z complemented main Sector C. Trench North, despite exhibiting different occupational evidence, resembled Sector C, although the stratigraphy showed only four occupational periods (Marcucci et al., 2014).

The same excavation, sampling, and retrieval procedures were employed in each of the settlement areas (Sectors A and C, and Trench North); a grid of 2 × 2 m excavation units was used for all excavations. Sediments were sieved with one to five mm mesh sizes. When stratigraphic units presented concentrations of dark colored anthropogenic deposits, a ca. eight litre basket of sediment was processed for flotation with 0.5–3 mm sieves. For the graveyard area (Sector B), sediments from all graves were sampled from the surface to the bottom of the pit with one mm sieves. In summary, all osteological remains were recovered in situ (directly from the excavation) or from sieves.

### Ra’s al-Hadd

Specimens retrieved from HD-6 discussed in this paper were collected during the 1997, 2000, 2002, 2007, and 2008 seasons. Excavations and sample retrieval at Ra’s al-Hadd followed standardized procedures, adapted to the contexts being investigated. Deposits from F/G Courtyard and Room 54 (Fig. 4) were excavated using 2 × 2 m excavation units; Room 33 was excavated using 50 × 50 cm units in order to analyze the distribution of small objects which were remarkably common in this space: beads and other evidence of bead and ring production, as well as *D. coriacea* ossicles, Anthropogenic deposits removed from excavations from within these enclosed spaces were collected in their entirety and dry-sieved using one to three mm mesh; artifacts were collected both in situ and by sieving. Samples retrieved from test trench T.T. 2/97 were collected in situ, and there was no systematic sieving. Likewise, sampling from outside dwelling areas was carried out in situ; nonanthropogenic deposits from these areas were only partially sieved (see Fig. 4 for a general plan of HD-6, which shows general locations and dimensions of discrete spaces relevant to this study).

Data concerning *D. coriacea* ossicles from both RH-6 and HD-6 discussed herein are original contributions to this paper, as is information concerning most of the retrieval contexts for HD-6 (i.e., Room 54, F/G Courtyard, T.T.2/97, and outside of buildings). The assemblage of artifacts from Room 33 at HD-6 has been addressed in a previous article (Azzarà, 2009: 7, figs. 5, 6.1, 6.4–5. 6.8).

The archeological materials described in this paper are stored permanently in the collections of the Department of Excavations and Archeological Studies of the Ministry of Heritage and Culture of the Sultanate of Oman in Muscat.

## Results

### Identification of *Dermochelys coriacea*

**Figure 5:**
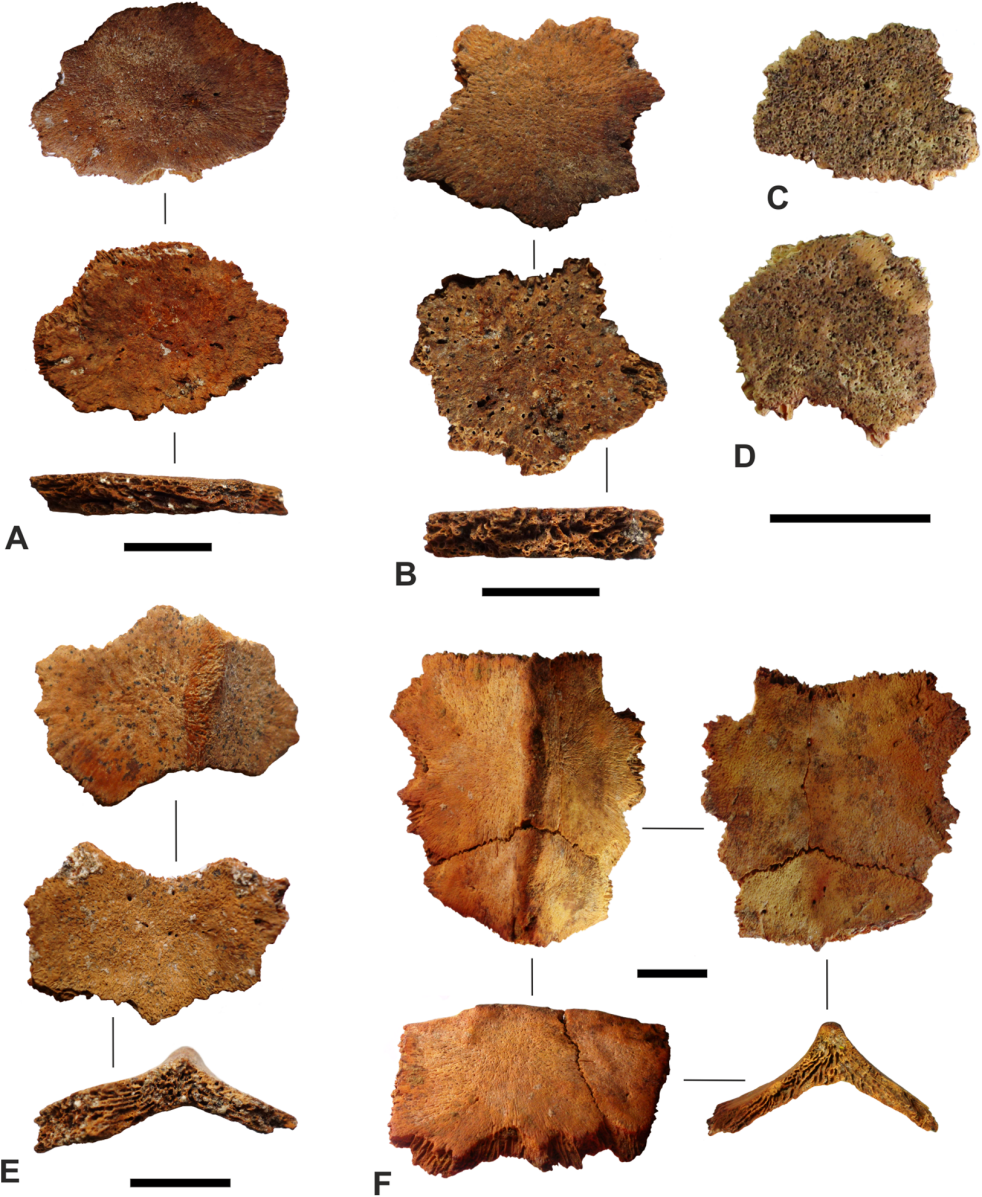
Examples of 6 ossicles of *Dermochelys coriacea* (Vandelli, 1761), recovered from the Sultanate of Oman, during the present study. (A) One of the largest ossicles encountered from Ra’s al-Hamra-6, from Sector C, test pit TT-Z, showing the flat (nonridged) external surface, the internal (or visceral) surface, and a side view. (B) A flat, nonridged, ossicle from Ra’s al-Hamra-6, Sector C, test pit TT-Z, showing: the flat (nonridged) external surface, the internal (or visceral) surface, and a side view. (C) A flat, nonridged, ossicle from Ra’s al-Hadd-6, Room 33, showing the external surface. (D) A flat, nonridged, ossicle from Ra’s al-Hadd-6, Room 33, showing the external surface. (E) A ridged ossicle from Ra’s al-Hamra-5, Sector C, test pit TT-Z, showing: the ridged external surface, the internal (or visceral) surface, and a side (anterior or posterior) view. (F) Two tightly articulated ossicles, the largest of which is one of the largest ossicles encountered from Ra’s al-Hadd-6, Room 54, showing the ridged external surface, the internal (or visceral) surface, a lateral view, and a side (anterior or posterior) view. Thin lines connect different views of the same ossicle. All scale bars equal 10 mm. Photos: M. Delfino.

**Figure 6:**
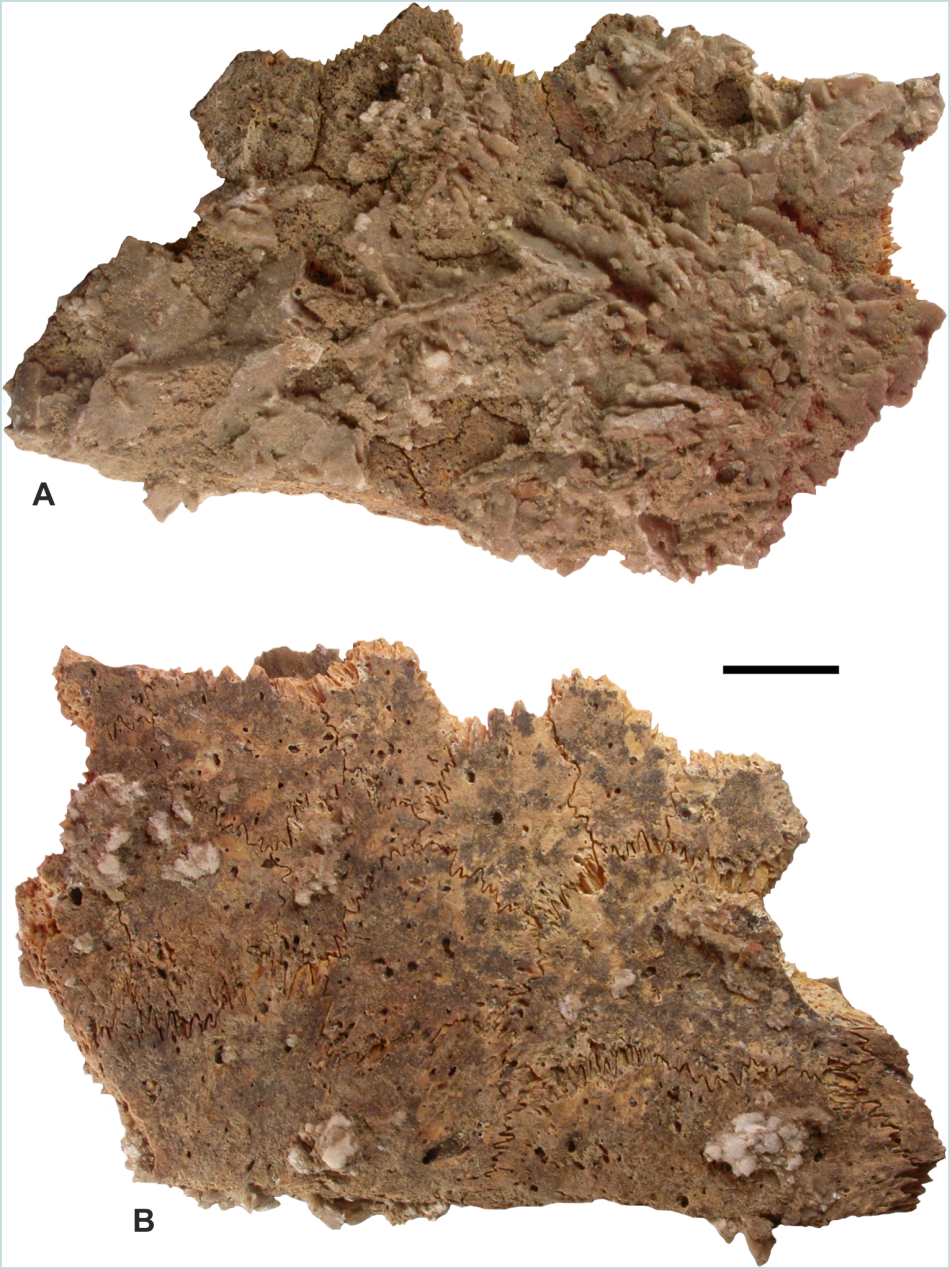
Remains of *Dermochelys coriacea* (Vandelli, 1761), recovered from the Sultanate of Oman, during the present study, excavated from Room 33 of the Bronze Age archeological site at Ra’s al-Hadd-6. This carapace fragment shows 14 ossicles articulated and covered with mineralized concretions. (A) Dorsal, exterior view. (B) Interior view. The scale bar equals 10 mm. Photos: M. Delfino.

Irregularly shaped bony ossicles were recovered from both sites: a total of 184 from RH-6, and a total of 149 from HD-6. These laminar structures are approximately round, elliptical, or polygonal, with borders covered by very fine, needle-like projections (Fig. 5). The maximum dimension across the lamina varies considerably, from a few millimetres to a few centimetres. One of the largest ossicles, from RH-6, Sector C, TT-Z, is 30 mm in the greatest dimension and 21 mm perpendicular to that; its dorsoventral thickness varies from three to four mm (Fig. 5A); one of the largest ossicles from Ra’s al-Hadd, Room 54, is shown in Fig. 5F. Two of the smallest ossicles, from RH-6, Sector A, are 9 × 7 × 3 mm and 9 × 5 × 3 mm. The surface of the ossicles that was toward the exterior of the animal is generally smooth or uniformly porous, whereas the visceral surface is irregular with sparse vascular foramina. The vast majority of ossicles, 99% from RH-6 and 89% from HD-6, are essentially flat (Figs. 5A–5D), while a small proportion of the ossicles are tectiform with a convex external surface topped by a more or less central ridge (Figs. 5E and 5F). Most of the ossicles were solitary, but in HD-6 Room 33 at least 14 were retrieved while still articulated with other ossicles, held together by the deeply interdigitating needle-like projections, and a fine mineralized concretion (Fig. 6).

**Figure 7:**
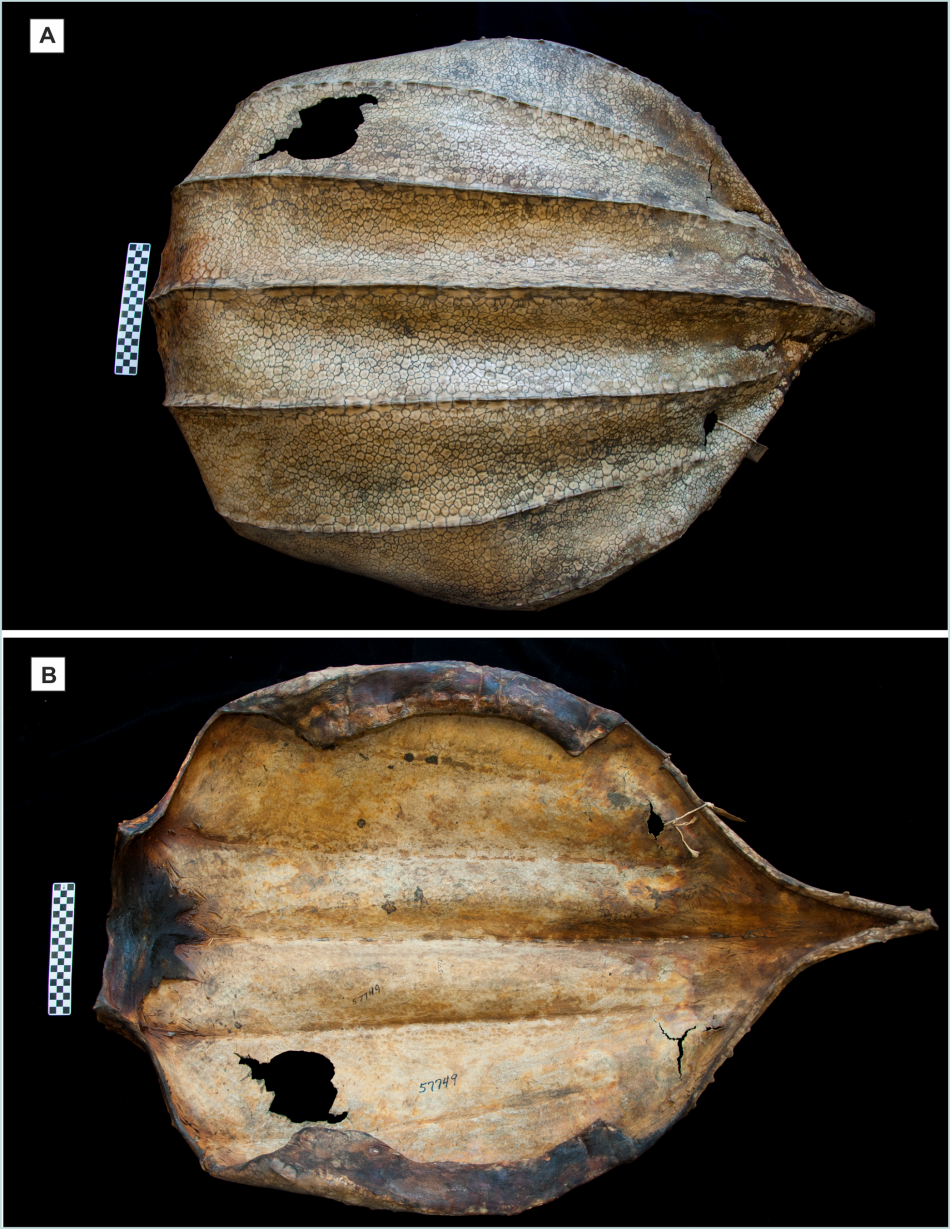
Desiccated carapace of *Dermochelys coriacea* (Vandelli, 1761). American Museum of Natural History (AMNH) specimen no. R 57749, received from aquarium in 1936, no further collection data are available; at the anterior end of the specimen the ruler with alternating black and white squares is 10 cm long, which shows that the specimen is approximately half the length of an adult. (A) Dorsal view showing the mosaic of thousands of small, irregular, interlocking ossicles that make up the dorsal shell. (B) Ventral view of the same specimen, showing the dried tissue that supports the ventral surface of the mosaic, and the nuchal element, at the extreme anterior (left side). Photos: L. Vonnahme.

The size and shape of the ossicles, along with their interdigitating sutures that interlock tightly with neighboring ossicles, as well as the tectiform structure of some ossicles, are diagnostic features of the carapace, and plastron, of most post-Eocene marine turtles of the family Dermochelyidae (Supplemental Information 1). The relative thinness of the 333 Omani ossicles and the deeply convex exterior surface of those ossicles that are tectiform, with a more or less central ridge and deeply concave visceral surface, and the abundant, fine needle-like projections covering the sides of the ossicles, are characters consistent with *D. coriacea* that contrast from the morphology of the extinct dermochelyid *P. polygonus*, whose ossicles are much thicker (very rarely less than five mm), virtually flat on the visceral surface, and lack the dense cover of fine needle-like projections on the sides (Delfino et al., 2013). The tectiform, ridged ossicles documented herein correspond to somewhere from one of the seven anteroposterior ridges of the carapace (less probably to one of the six longitudinal ridges of the plastron); the flat ossicles correspond to the broad areas between longitudinal ridges (Wood et al., 1996; Frazier, Gramentz & Fritz, 2005; Delfino et al., 2013; see also Fig. 7).

There are no obvious differences between ossicles retrieved from RH-6 and those from HD-6. No cut marks, signs of burning, or other indications of postmortem modification were observed on any of the ossicles. The interlocking ossicles of this turtle form the majority of the carapace, or dorsal shell (Fig. 7), and part of the plastron, or ventral shell. No other bones (i.e., no cranial, axial, appendicular, or thecal elements from the carapace or plastron) were identified as, or suspected to be from, *D. coriacea* from either RH-6, HD-6, or any other site in Oman.

### Ra’s al-Hamra-6

The majority of the 184 ossicles recovered from RH-6 (Table 1) came from settlement areas (91%), showing that all the sectors excavated, and nearly the entire occupational sequence that has been sampled, yielded remains of *Dermochelys*: 71 ossicles from Sector A; 72 from Sector C; and 25 from Trench North. Of the six funerary structures that were excavated during 2012 and 2013 in Sector B, only Grave 7 (without any human skeleton and thought to be a symbolic burial structure) produced ossicles, of which 16 were recovered. Out of the 184 *D. coriacea* ossicles recovered from RH-6 only two (1%) were ridged, both from Sector C, Period V (Table 1: collection 26).

**Table 1:** Ossicles of *Dermochelys coriacea* (Vandelli, 1761), from Ra’s al-Hamra-6.

Collection no.	Number of ossicles		Sector	Stratigraphic unit(s)	Period	Figure 5
	Total	Ridged only				
1	11		A	SU109	A-II	
2	1		A	SU19	A-II	
3	32		A	SU11	A-II	
4	25		A	SU3	A-II	
5	2		A	SU2	A-II	
6	3		C	SU118	C-V	
7	1		C	SU103	C-V	
8	3		C	SU98	C-V	
9	14	2	C	SU97	C-V	E
10	1		C	SU91	C-V	
11	8		C	SU90	C-V	
12	4		C	SU89	C-V	A
13	6		C	SU75	C-V	
14	5		C	SU73	C-VI	B
15	26		C	SU57	C-VI	
16	1		C	SU3	C-VI	
17	1		Tr N	SU55	TN-I	
18	6		Tr N	SU49	TN-I	
19	9		Tr N	SU31	TN-I	
20	2		Tr N	SU25	TN-I	
21	1		Tr N	SU24	TN-I	
22	5		Tr N	SU22	TN-II	
23	1		Tr N	Surface	TN-IV	
24	16		B	SUs 12 and 13	Grave 7	
Total	184	2				

**Note:**

Collection no. = the arbitrary serial number that corresponds to the collection of ossicles that were recovered from specific stratigraphic units in a certain locus; Number of ossicles = the number of ossicles recovered from a collection; Total = number of ridged + nonridged ossicles recovered from a collection; Ridged only = number of ridged ossicles recovered from a collection; Sector = the sector in RH-6 (see Marcucci et al., 2014 for details). All collections were from settlement contexts, with the exception of collection 24 from Grave 7; Stratigraphic unit(s) = the stratigraphic unit(s) designated during the excavation from which ossicles from this collection were recovered: SU98 of Sector C corresponds to Pit 5; SUs 12 and 13 of Sector B correspond to Grave 7; Period = the occupational period to which the stratigraphic unit(s) in this sector have been designated: Sector A has II Periods; Sector C has VI periods; Trench North has IV Periods; Figure 5 = the image in Fig. 5 which illustrates an ossicle from this collection.

The 71 ossicles found in Sector A (Table 1: collections 1–5) are all from Period II of this sector, the locus with the oldest known signs of human presence at RH-6. These ossicles were primarily associated with anthropogenic deposits and structural features located a few centimeters above the bedrock, in a very restricted area between traces of two habitational structures. The 72 *D. coriacea* ossicles unearthed in Sector C are from later occupations, Periods C-V and C-VI; the artifacts and ecofacts from these periods indicate a larger human settlement and diversification of subsistence activities at the site. The ossicles, often associated with traces of food-processing and a diversity of artifacts (including worked shells, shell fish hooks, shell-beads, gorges, bone tools, and stone tools), were discovered in a variety of depositional contexts: sandy sediments containing well-preserved fish bones in situ (Table 1: collections 10 and 13), over “living” floors (Table 1: collections 6, 7, and 15), and from a large pit (Table 1: collection 8). All but six of the 25 ossicles recovered from Trench North came from the lowest stratigraphic levels of this trench. These ossicles laid on tabular gravelly surfaces (Table 1: collections 19 and 22) and within compacted deposits of fish bones or seashells (Table 1: collection 17), bearing traces of ephemeral settlement structures (pits and post holes; Table 1: collections 18, 20, and 21). Grave 7 (probably a cenotaph) produced 16 ossicles from the filling of the pit (Table 1: collection 24). These were mixed with many fragmented mollusc shells that were commonly found in the general surroundings outside the grave, and whose presence in the grave is therefore probably incidental to the action of filling.

### Ra’s al-Hadd-6

Of the 149 ossicles recovered from HD-6, 130 (87%) were found in contexts ascribed to Phase I-3; another 13 (9%) were from contexts attributed to Phase I-4, and just six ossicles were found in a context attributed to Period II (Table 2). A total of 16 (11%) of the 149 ossicles from HD-6 were ridged. Most of the *D. coriacea* remains from HD-6 were collected from occupational deposits. A total of 94 ossicles (63%) was retrieved from an indoor space, 2.3 × 1.5 m, Room 33 (Fig. 4), from deposits ascribed to Phase I-3b (Table 2: collection1); none of these were ridged. This room showed evidence of workshop activities related to the production of shell and stone jewelry. Two ossicles, both ridged, came from a similar context, Room 54, but assigned to Phase I-4a (Table 2: collection 6). In addition, 35 ossicles (23% of the Ra’s al-Hadd total), were collected in the F/G Courtyard, Phase I-3c; eight of these were ridged. This was the second largest number of specimens from a single locus, and the largest proportion of ridged ossicles (23%) from any single recovery location. These ossicles from F/G Courtyard were together with mixed plant and animal remains related to food-preparation activities (Table 2: collections 2 and 3). An additional three ossicles were recovered from the same locus, but a later occupation, Phase I-4a (Table 2: collection 7); none were ridged. A total of eight ossicles were from mixed sediments indicative of the abandonment of the settlement (or a part of it) that occurred between occupational phases, namely at the end of Phase I-3c and beginning of Phase I-4b (Table 2: collections 4 and 8); three of these were ridged. One ridged ossicle was recovered from outside of buildings, Phase I-4a (Table 2: collection 5). Six more ossicles came from the late occupation of the area, T.T.2/97, of which two were ridged (Table 2: collection 9).

**Table 2:** Ossicles of *Dermochelys coriacea* (Vandelli, 1761), from Ra’s al-Hadd-6.

Collection no.	Number of ossicles		Locus	Stratigraphic unit(s)	Phase	Figure 5
	Total	Ridged only				
1	94		Room 33	SUs 791, 794, and 797	I-3b	C and D
2	34	8	F/G Courtyard	SU2049	I-3c	
3	1		F/G Courtyard	SU2050	I-3c	
4	1		Outside of buildings	SU2071	I-3c	
5	1	1	Outside of buildings	SU2097	I-4a	
6	2	2	Room 54	SU2057	I-4a	F
7	3		F/G Courtyard	SU2189	I-4a	
8	7	3	Outside of buildings	SU2190	I-4b	
9	6	2	T.T.2/97	SU19	II	
Total	149	16				

**Note:**

Collection no. = the arbitrary serial number that corresponds to the collection of ossicles that were recovered from specific stratigraphic units in a certain locus; Number of ossicles = the number of ossicles recovered from a collection; Total = number of ridged + nonridged ossicles recovered from a collection; Ridged only = number of ridged ossicles recovered from a collection; Locus = the location at HD-6: see Azzarà (2009) for details regarding “Room 33”; for “F/G Courtyard” see Azzarà & Cattani (2007: 5–7, figs. 5 and 7) ; for “Outside of buildings” see Azzarà & Cattani (2007: 2–3) for “Room 54” see Azzarà (2015: 180); for “T.T.2/97” see Tosi & Cattani (1997: 1); Stratigraphic unit(s) = the stratigraphic unit(s) designated during the excavation from which ossicles from this collection were recovered; Phase = the occupational phase (or subphase) to which the stratigraphic unit(s) in this locus have been designated. Phase I-3c has been dated to 2,880–2,570, 2σ cal BC (Azzarà, 2013: fig. 3); Figure 5 = the image in Fig. 5 which illustrates an ossicle from this collection.

## Discussion

This paper is the third ever published account of *D. coriacea* remains from archeological sites, from any locality, from any period, but since very little has been explained about the stratigraphic and archeological contexts of the previous two records, there are few points of comparison to make or precedents that can be followed. The two previously published records are from the Caribbean (Frazier, 2005), and much later dates: Cerro Brujo, Bocas del Toro, Panama (600–900 AD) (Wing, 1980: table 1) and St. Thomas, US Virgin Islands (300–700 AD) (Wing, DeFrance & Kozuch, 2002); other than documentation of this species, these earlier records provide next to no information on the remains of *D. coriacea* that were recovered. Nonetheless, several questions about, or stemming from, the Omani *D. coriacea* ossicles warrant exploration, several of which must be addressed in a subsequent study:

1. Are the ossicles reported herein of archeological relevance, or simply beach debris mixed in with archeological materials?
2. How many individual *D. coriacea* were involved in the RH-6 and HD-6 records?
3. Why are there not more prehistoric records of *D. coriacea* from SE Arabia or anywhere else in the world?
4. Why were only ossicles recovered, with no other bones (e.g., skull, vertebral column, larger thecal bones from the carapace or plastron, or axial skeleton) reported?
5. Were environmental conditions off the coast of Oman during the Mid-to Late Holocene more favorable to *D. coriacea* than they are today?

### Are the ossicles reported herein of archeological relevance, or simply beach debris mixed in with archeological materials?

One interpretation of the ossicles reported herein (as proposed by a reviewer) could be that they were simply the remains of *D. coriacea* that had washed up on beaches, decomposed, and became miscellaneous components of beach debris. This explanation requires these animal remains to then be incorporated into diverse inland stratigraphic sequences, from different time periods, after which they would be found in close spatial and temporal association with other materials identified as cultural artifacts (e.g., worked shells, fish hooks, gorges, net weights, shell and stone beads, bone and stone tools, post holes, walls, etc.) as well as associating with ecofacts (e.g., dense accumulations of fish bones and mollusc shells, including concentrations of marine animal remains that had been modified by burning, boiling, and/or other forms of cooking and modification by human agency). In this regard it is essential to understand the details of the strategic and temporal contexts in which the ossicles were found, as summarized above in the Results section. These details show that at both RH-6 and HD-6 the vast majority of ossicles were distant from beaches where miscellaneous debris would have been deposited. Moreover, at least 90% of the ossicles were clearly in archeological contexts, including close spatial association with both diverse cultural artifacts and diverse ecofacts, as well as the occurrence on occupational floors (i.e., primary evidence related to actual human action), and presence in relatively small closed spaces, enclosed by solid walls, which were evidently still in use at the time of the deposit. Finally, there were no significant macroscopic signs of abrasion or tooth marks on any ossicles that would have resulted from wind, tidal, or scavenger transport.

Animal remains found in archeological contexts, which show no macroscopically visible signs of carnivore damage, are regularly considered to be evidence of past human predation, if not capture, of the species in question (Henshilwood et al., 2001). Nonetheless, empirical evidence that shows human modifications of prey remains is clearly preferable (Thompson & Henshilwood, 2014). In this light, although 333 ossicles of *D. coriacea* were found in different archeological contexts at RH-6 and HD-6, it has been noted no macroscopic signs of human modification were found on these specimens (i.e., no signs of cut marks and no signs of burning/charring).

The usual, commonly recognized signs, and evaluation protocols, for mammalian bones can be of little relevance for chelonian remains, a point amply explained by Thompson & Henshilwood (2014). Furthermore, various signs of modification by human agency may be missed unless detailed microscopic analyses are carried out; yet, even with comprehensive microscopic analysis of animal food remains, clear signs of modification by human agency may be rare (Thompson, 2010). For example, Thompson & Henshilwood (2014) did an exhaustive analysis of 4,343 bones, primarily of the small angulated tortoise, *Chersina angulata* (Schweigger, 1812), which is commonly prepared by roasting entire on the fire. Although they reported charring on over 66% of the sample, the incidence of cut marks was only 1%, percussion marks were recorded on less than 2% of the sample, and “tooth marks” (not necessarily all attributable to humans) were observed on 5% of the sample. Because no microscopic analyses of the RH-6 or HD-6 ossicles were undertaken, we cannot assert that there are no signs of modification by human agency on these remains.

Moreover, evidence of human modification to faunal remains is not a requisite to establish an archeological context, but rather it is an additional, compelling source of evidence. In the specific case of the present study, the production of conspicuous cut marks on relatively small bones that form a large mosaic on the shell of an enormous animal, such as *D. coriacea* ossicles, seems highly unlikely. Hence, it is concluded that the vast majority of the Omani ossicles clearly pertain to archeological contexts (see Supplemental Information 4 for more details).

### How many individual *Dermochelys coriacea* were involved in the RH-6 and HD-6 records?

The ossicles of *D. coriacea* do not provide a means to distinguish among age classes or sexes, much less individuals; hence, the usual method of calculating minimum number of individuals (MNI) (Grayson, 1979: 205 ff), dependent on morphological characteristics, is not feasible. Therefore, to comply with this standard procedure for estimating MNI, that has been practiced in zooarcheological research for decades, the “maximum distinction method” (Grayson, 1973: 433) was used to establish spatial-temporal boundaries, by which aggregates of ossicles could be distinguished. This yielded estimates of between six and 24 individual *D. coriacea* from RH-6, and between four and nine individuals from HD-6 (see Supplemental Information 5 for details).

Radiocarbon investigations indicate that the Omani ossicles were deposited between about 5,600 and 2,600 BC, a span of about 3,000 years. The ossicles from just RH-6 would have been deposited between about 5,600 and 3,080 BC, yielding a rough average minimum deposition rate (RAMDR) of no less than one *D. coriacea* every 300 years, and as many as one every 75 years.

Ossicles from just HD-6 would have been deposited between about 3,100 and 2,600 BC, indicating a RAMDR of no less than one *D. coriacea* every 125 years, and as many as one every 55 years. However, such comparisons of deposition rates between sites are based on the assumption that volumes of substrate sampled at the sites under study are comparable, but this information is not available.

Finally, numbers used for estimating the MNI—however, they may be obtained—represent only the estimated MNI recovered from the sampling under study. They cannot pretend to estimate the actual number of individuals deposited at the site.

### Why are there not more prehistoric records of *D. coriacea* from SE Arabia or anywhere else in the world?

For a vertebrate animal with a circumglobal distribution, distinctive morphology, and large body size, it is remarkable that there is so little archeological and paleontological information on *D. coriacea*: just three published archeological records (including this one), and the general belief that there are no fossils (see Introduction). A lack of adequate prehistoric technologies, and/or cultural aspects (e.g., taboos) that reduced the chances of capturing and depositing even parts of these turtles in middens or habitational areas could explain the lack of archeological records, but it is unlikely that these factors could explain the scarcity of records for more than seven millennia. And, these considerations do not explain the lack of fossils. A central question appears to be taphonomic.

A possible explanation for the lack of reported remains could be that the relatively small, irregularly shaped ossicles have been missed, and simply discarded in the overburden. However, since sieving with meshes no larger than five mm has been standard practice for archeological sites in this area, it is close to impossible that these obvious, although perhaps unusual, bones would not be noticed, for even the smallest ossicles would not pass through a five mm mesh. Moreover, much smaller, more delicate, fish bones have been regularly collected in very large numbers from these same sites. The challenges of collecting and identifying small irregular bones, such as ossicles of *D. coriacea*, are not trivial; these apparently worthless specimens can easily be unknown, overlooked, and undervalued. For example, it is notable that *D. coriacea* ossicles at RH-6 have been documented only during the most recent 2012 and 2013 campaigns, when ossicles were widely distributed horizontally and stratigraphically. Remarkably, this was the first time this distinctive species had been documented at RH-6, even though work began there in 1975 and was continued for almost 40 years before the species was reported. Indeed, even other nearby sites at Ra’s al-Hamra, RH-5 in particular, but also RH-4 and RH-10, which has been extensively worked starting in 1981, have produced no known *D. coriacea* material. Possibly, detailed re-evaluations by faunal specialists of remains recovered from earlier excavations could detect ossicles where they were previously overlooked, undervalued and/or unknown, for even very experienced and thorough zooarcheologists would not have expected a species that is virtually unknown from archeological sites (M. Uerpmann *in litt.* to JF November 12, 2017; see also Hoch, 1979: 596).

The enigma is further emphasized by the fact that hundreds of archeological sites are known along coastal Oman, and farther north, and in some sites excavations have been conducted for years—if not decades (Frifelt, 1995; Cleuziou & Tosi, 2000; Charpentier, Marquis & Pellé, 2003; Beech, 2004; Charpentier, 2008: fig. 1; Cavulli & Scaruffi, 2012; Berger et al., 2013: 3091; Charpentier et al., 2013). There are no explicit records, but the amount of sediment that has been examined from just a single site may be several tonnes, and considering all coastal sites that have been worked, many tonnes of sediment will have been sieved since the 1970s. Yet, the only recorded *D. coriacea* remains are those reported herein.

Nonetheless, even if a site has been investigated for years, this is no guarantee that the faunal remains have been adequately studied; the fact that *D. coriacea* ossicles were reported at RH-6 for first time nearly four decades after excavations began, is a case in point. Few sites in the region, known for long-term archeological research, have benefited from systematic, thorough faunal studies. Even sites that have been extensively worked and included faunal analysis (such as RH-4, RH-5, and RH-10) could ultimately yield *D. coriacea* remains after more thorough investigations of faunal materials; for example, in their detailed study of numerous sites at and near Ra’s al-Hamra, Uerpmann & Uerpmann (2003: 200; M. Uerpmann *in litt.* to JF November 12, 2017) explained that they did not have access to all the faunal remains that had been recovered. Hence, the paucity of records could be explained, at least in part, by the lack of systematic zooarcheological analysis; and even when faunal analyses have been carried out, there may have been instances when the researcher was unfamiliar with the irregularly shaped and seemingly unusual ossicles, that are in fact ready identifiers of *D. coriacea*.

Another way of considering the lack of archeological records of *D. coriacea* is to ask the opposite question: what was it about RH-6 and HD-6 that makes these sites so unusual that they yielded remains of animals almost never reported? Cleuziou & Tosi (2007: 57) provide a clue: “…by the beginning of the fourth millennium BC the settlements of the Arabian Sea coast displayed all the evidence of a new and revolutionary adaptation that took place in very few areas across the globe: deep sea fishing.” In other words, the environmental conditions in which RH-6 and HD-6 existed made possible unique opportunities for interactions with deep sea marine animals—which include *D. coriacea.* Hence, where human settlements were not close to the deep sea there was less probability that such sites would yield remains of this pelagic animal. Nonetheless, this proposition alone does not explain the absence of records from other sites along the coast of the Arabian sea, such as as-Suwayh, al-Khabbah, and Ra’s al Jinz (Fig. 1). It will be necessary to wait until systematic zooarcheological investigations at these sites produce more robust data about their respective faunal records.

As regards fossils, some vertebrate palaeontologists have observed that “Pelagic taxa with thin fragile shells (e.g., *D coriacea*) don’t make for good fossils” (J. Parham *in litt.* to JF September 20, 2017; also R. Hirayama *in litt.* to JF September 11, 2018). It does seem that taphonomic aspects are central to this enigma. As Wood et al. (1996: 266) explained: “when a beached leatherback dies, its shell does not long retain the rotund profile characteristic of live individuals. The carapace first sags and then quickly collapses into a jumble of disarticulated ossicles—a true paleontologist’s nightmare.” Although this description is certainly accurate, even a “jumble,” and haphazard scattering, of small bones is nothing unusual for the experienced zooarcheologist or palaeontologist. Nonetheless, there is no simple explanation for the remarkable rarity of archeological and fossil records of *D. coriacea*.

### Why were only ossicles, and no other bones, recovered from RH-6 and HD-6?

The present study documents 184 ossicles from Ra’s al-Hamra site 6, and 149 ossicles from Ra’s al-Hadd site 6: a grand total of 383 ossicles. Remarkably, no other bone from *D. coriacea* has been reported from either of the two sites where ossicles were recovered, bones which evidently derived from, at least, between 10 and 33 individual turtles. The only other published records of this turtle from archeological sites are Cerro Brujo, Bocas del Toro, Panama (Wing, 1980: Table 1) and Tutu Main Street, St. Thomas, US Virgin Islands (Wing, DeFrance & Kozuch, 2002), which are based on limb bones from 3, or 4, individual turtles from Panama, and a single carpal, or tarsal, bone from St. Thomas (unpublished species cards and reports on file at Florida Museum of Natural History, Environmental Archaeology Program, FLMNH-EA accession numbers Cerro Brujo Acc# 0152, Tutu Acc# 0489).

The conditions in which bony remains were deposited in many coastal sites in Arabia indicate that preservation conditions were very good. As Beech, Charpentier & Méry (2004: 331) explained: “[d]ue to the presence of large quantities of marine shells at these sites, the relatively high calcium carbonate content ensures that animal bones are much better preserved within these ‘shell-middens’” (see also Hoch, 1979: 595; Marcucci, 2014: 506). The general appearance of many bony remains (particularly marine turtle, most likely *Chelonia mydas*) from RH-6 and HD-6 do not indicate serious problems of deterioration, although in some cases there is evidence of some deterioration, from unknown causes. However, even if there were widespread deterioration of turtle bones, this alone would not explain why the only remains of *D. coriacea* that have been recovered from Oman are ossicles.

Ossicles of *D. coriacea* may be more compact and resistant to deterioration than other bones of this animal, and therefore more likely to endure, especially for millennia. Studies of bone morphology of this turtle—particularly osteochondral development—show that the limb bones are highly vascularized with large cartilaginous portions remaining throughout life, and not composed of dense periosteal bone, as occurs in other marine turtles (Rhodin, Ogden & Conlogue, 1981; Rhodin, 1985; Snover & Rhodin, 2008). Although these larger bones appear to be hefty and solid, their structure would lend them more susceptible to environmental deterioration than bones that have thick layers of dense periosteal bone. In contrast: “…ossicles are epidermal ossifications of much denser, less vascularized bone, than the appendicular, cranial, or spinal elements, which are more porous with less sclerotic compact bone, and the ossicles would therefore survive more easily in middens.” (A. Rhodin *in litt.* to JF October 2, 2017) (for details of ossicle microstructure see Delfino et al., 2013: 774). This indicates that the bones that one might normally expect to find—limb, axial, and cranial bones—are more highly subject to taphonomic degradation than are ossicles, particularly over periods of centuries and millennia. One way or the other, J. Parham notes (*in litt.* to JF September 20, 2017) that the majority of Dermochelyid fossils are ossicles, with no other bones. Hence, both zooarcheologists and paleontologists need to be aware of the limitations of finding skeletal remains of these turtles, a sentiment reinforced by Hirayama (*in litt.* to JF September 24, 2018).

### Were environmental conditions off the coast of Oman during the Mid- to Late Holocene more favorable to *Dermochelys coriacea* than they are today?

Present-day patterns of abundance and distribution of *D. coriacea* most likely do not reflect the biogeographic situations when RH-6 and HD-6 were inhabited. This point has been made by various authors: “marine transgressions or regressions in the relatively shallow waters of the Gulf must have influenced the life cycles of migrating fishes [and other marine organisms], so in this case we perhaps cannot transfer present day observations back into a fourth-third millennia context.” (Biagi et al., 1984: 50). Indeed, over the past 20,000 years the Gulf area has experienced enormous changes, evolving from a virtually dry area to a flooded sea by about 6,000 BP; at present times it is almost an inland sea with an average depth of about 35 m (Lambeck, 1996).

However, the specific accounts of past conditions vary—sometimes considerably—between different studies. “At 6,000 year BP the predicted sea levels [in the Gulf of Oman] lie above the present level by about two to three m.” (Lambeck, 1996: 47), and “…levels along this section of the coast [the northern Gulf] having been one to three m above the present levels between about 3,500 and 6,000 year BP.” (Lambeck, 1996: 48; see also details in his Table 1). In contrast, Zarins (1992: 57) reported that “[b]y 5,000 BC sea-level was at −10 m of present-day m.s.l. …and the sea-coast was approximately 100–150 km from it present-day position…with the modern shoreline returning in stages between 2,000 and 1,000 BC”; and “[b]etween 5,000 and 2,000 BC sea level may have risen as much as three m above [present-day] m.s.l. …” There are numerous challenges to interpreting evidence relating to past conditions (Lambeck, 1996), so it is not surprising that accounts of different scenarios have been proposed.

Nevertheless, there is clear evidence of a significant marine transgression from Kuwait to Oman, and on to the Red Sea, with different phases of expansion and contraction of the Gulf between 5,000 and 2,000 BC. Because of different environmental conditions between 5,000 and 3,000 BC, the summer Southwest Monsoon, whose latitudinal position is determined by the Inner Tropical Convergence Zone, is thought to have been as far north as central Arabia and present-day southern Iraq, “…during which the entire Gulf was a dry plain filled with river runoff from Mesopotamia, Arabia and Iran. Gradual marine infilling began by 4,000 BC which culminated in a higher sea-stand by 3,000 BC” (Zarins, 2008: 218).

Along the Oman coast, from 6,000 to 3,000 cal BC, five cycles of sea level rise and fall are recognized, associated with other environmental changes including mangrove development and salt flat expansion. When RH-6 was occupied, about 5,600–4,500 BC, sea level and landscapes are thought to have been similar to the present, although at the end of the RH-6 occupation period, beginning about 4,800 BC, “the maximum postglacial sea transgression (+2/3 m)” was reported (Berger et al., 2013: 3096). Later, at the time when the RH-6 cemetery was in use, around 4,000–3,800 BC, sea level and mangrove development were decreasing. By the time HD-6 was occupied, around 3,000 BC, sea level was again falling and extensive salt flats (“sabkhas”) were developing (Berger et al., 2013). Obviously, these major differences between past and present in sea level, marine shorelines, as well as the surface area and depth of the Gulf would be intimately related to the distributions and abundance of marine turtles during these periods.

In this respect, Dutton et al. (1999) reported singularly low genetic diversity (mt DNA) in their global sample from 10 different nesting grounds of *D. coriacea*, which they hypothesized was due to a global reduction in the population, caused by global cooling during the Plio-Pleistocene. They further suggested that later, during the Holocene, global warming enabled relatively recent population expansion and global population radiation. This scenario suggests that Mid-Holocene populations of *D. coriacea*, likely occurring off the coasts of Oman, were expanding relatively rapidly.

There are two major aspects of past environments for RH-6 and HD-6 that are especially relevant: beaches where *D. coriacea* may have nested, and coastal areas, particularly deep waters, where these turtles could have occurred during seasonal feeding migrations. Both beach and marine aspects are interrelated, but it is simplest to treat them separately.

It is not known if steeply sloping sand beaches–characteristic of *D. coriacea* nesting beaches (Frazier, Gramentz & Fritz, 2005: 285; Eckert et al., 2012: 28–29)–were more, or less, available during any of the four periods discussed above (i.e., main RH-6 occupation, end of RH-6 occupation, RH-6 graveyard in use, and HD-6 occupation). Nonetheless, changes in sea level would have direct effects on coastal and beach physiognomy. Where steep cliffs characterize present-day coasts, such as at Ra’s al-Hamra on the Batinah coast (Durante & Tosi, 1977: 141), sea level fall could have resulted in greater availability of potential *D. coriacea* nesting beaches. Hence, at the end of the RH-6 occupation and when the RH-6 graveyard was in use, there could have been increased *D. coriacea* nesting within the vicinity of the site; the same could have been true when HD-6 was occupied.

Durante & Tosi (1977: 141) reported that until the late 1960s turtles (species not identified, but most likely *Chelonia mydas*) nested on “the long Ra’s al-Hamra beach”; Uerpmann & Uerpmann (2003: 179, 204) pointed out that this long “flat sandy” beach is nearly three km west of the archeological site, and there are other small (“pocket”) beaches that are to the east, between cliffs of the actual headland (Fig. 2). They (2003: 204) suggested that the long beach to the west “probably did not exist during the time of occupation of RH 5, when there was a lagoon where the mangrove area is now.” The lack of evidence for steeply sloping beaches in the Ra’s al-Hamra area indicates that few, if any, *D. coriacea* nested there. With regard to Ra’s al-Hadd, there is a major nesting beach for *Chelonia mydas*, but, once again, these beaches do not seem to have the steep sloping profile characteristic of *D. coriacea* nesting beaches. Nonetheless, environmental—particularly sea level—changes during certain periods between 5,600 and 2,500 BC could have resulted in more nesting beaches for *D. coriacea* than are now known for this area, and hence increased the chances of remains of this turtle being deposited in these sites. An understanding of past availability of *D. coriacea* nesting beaches requires specific, detailed paleogeomorphological studies along the Batinah and Ja’alan coasts.

In addition to the question regarding possible past *D. coriacea* nesting on the coast of Oman, it is necessary to understand past abundance and distribution of these turtles in coastal waters. Although this reptile is generally regarded as a pelagic, open-ocean species, at least during modern times it is well known to occur in coastal waters in many parts of the world (Frazier, Gramentz & Fritz, 2005: 269–271, 286 ff; Eckert et al., 2012: 63–64, 71, 73, 81). This exception to an open ocean existence is tied to seasonal feeding migrations into certain coastal areas, particularly where upwelling results in fertile feeding grounds. As mentioned above, during modern times, the headlands of specific relevance to the present study—Ra’s al-Hamra and Ra’s al-Hadd—are well known for their seasonal upwellings, and thus, their rich fishing areas not far off shore. Schott & McCreary (2001) and Schott, Xie & McCreary (2009) have provided abundant oceanographic documentation for this phenomenon; indeed, the “Ras al Hadd Jet” figures prominently in their figures and discussions about oceanic currents.

Hence, during Mid- to Late Holocene the abundance and availability of these turtles in Omani waters, particularly off the Ja’alan and Batinah coasts, may well have been very different—perhaps more common—than during modern times. This raises the possibility that during certain periods within this span of three millennia remains of these turtles were more likely to be deposited at Neolithic and Bronze Age sites than they are today in Oman. If the amount of sediment sampled at RH-6 was comparable to the amount sampled at HD-6, the RAMDR of *D. coriacea* was evidently higher at HD-6 than at RH-6. This would indicate that conditions around 3,000 BC off the Ja’alan coast may have been more favorable for these turtles than they were around 5,000 BC off the Batinah. If there were significant environmental and/or socio-cultural differences between the two sites this would also have profound implications on differences in marine fishing, settlement characteristics, trade, and other activities between RH-6 and HD-6, much of which should be discernible from archeological evidence. As Lambeck (1996: 555–556) explained, the key to understanding a multitude of questions about how past societies in this region developed, functioned and survived—their economies, technologies, and relationships with their surroundings—is understanding past environmental conditions and their changes through time.

## Conclusions

The results of the present study reveal far more than a new record of a species rarely documented from archeological sites. They provide clues about the past, and raise questions about both pending and new issues related to the geographic area, sites, communities, turtles, and environments under study, as well as to much broader applications of data that are required to illuminate these issues. Several major topics, developed in the preceding Discussion section, warrant emphasis.

A total of 383 ossicles of *D. coriacea* were recovered from two coastal sites in Oman, with dates that span from approximately 5,600 to 2,500 BC. At Ra’s al-Hamra 6 a total of 184 ossicles was recovered. All of these were found at least 600 m inland from the present shoreline, and at least seven m above the present highest level reached by the tide. At least 91% of the RH-6 ossicles were closely associated with either cultural artifacts and/or ecofacts resultant from the past occupation of the site. At Ra’s al-Hadd 6 a total of 149 ossicles were recovered; all of these were at least 200 m inland from the present shoreline. Of the HD-6 ossicles, 90% were recovered from closed spaces, as well as in association with cultural artifacts and/or ecofacts having been left from past human activities. Hence, nearly all, if not all, of the ossicles reported herein are interpreted to be archeological in origin, and not simply miscellaneous beach debris. Beyond that, however, there is no incontrovertible proof of how prehistoric humans interacted with the species; for example, no sign of modification through human agency was detected for any of the 383 ossicles.

Considering details of locus and stratigraphy from where ossicles were recovered indicates that the remains were deposited in multiple spatial-temporal events. Although there is no known way to distinguish ossicles between individuals of this species, or even between different sexes, a judicious use of excavation data to establish spatial-temporal groupings (“aggregations”) of the animal remains permits defensible estimates of MNI: between six and 24 turtles from RH-6 and from four to nine at HD-6. This approach warrants further application in other studies of zooarcheological specimens, for which there is little opportunity to make morphological comparisons between retrieved bones, but adequate archeological details from excavations are available.

The present study is only the third case of archeological documentation of this globally distributed, large, distinctive animal. Similarly, there are no known fossils, with the possible exception of one or two ossicles. Hence, taphonomic issues, likely the reason for this lack of remains, need to be further elucidated. Nonetheless, a plausible explanation for the paucity of records is that certain skeletal elements (i.e., ossicles) of this animal are subject to being overlooked, undervalued, and/or unknown. Hence, there is a pressing need for more systematic, detailed studies of faunal remains in this region.

It is also remarkable that the skeletal elements that are reported in both zooarcheological and paleontological research are, with very few exceptions, ossicles, despite the fact that the skeleton of this turtle includes scores bones other than ossicles, most of which are larger than ossicles. A possible explanation for this apparent anomaly is that ossicles are much more compact and resistant to environmental degradation than other bones, some of which include large proportions of cartilage and relatively little hard, resistant periosteal bone—even in adults. If that explanation holds widely throughout the family Dermochelyidae, it may help to illuminate topics that are often obscure to paleontology, particularly osteochondral development and bone composition in extinct species. With a similar focus, comparisons between the ossicles of the extinct Mid-Miocene turtle *P. polygonus*, and ossicles of the living *D. coriacea* (Delfino et al., 2013: 778), supported inferences about the diving habits of the long extinct animal. The detailed chondro-osseous work on appendicular elements (Snover & Rhodin, 2008) needs to be extended to the epithecal ossicles of this unique living turtle.

Beyond issues about methods for estimating MNI, taphonomic concerns, and skeletal characteristics of the animals under consideration, the documentation of a few hundred bony ossicles raises basic questions about past environments and how they may have impacted both the human communities and turtles that are central to this study. During modern times in Omani waters, *D. coriacea* has been rare, and often regarded as a vagrant, yet during past millennia remains of this turtle were repeatedly deposited in different strata of two distant sites. The study of past climates, sea levels, and oceanographic current systems, and how these major drivers may have affected beach physiognomy and upwelling areas along the Omani coast, have a particular relevance for an understanding of the relationship between prehistoric populations and marine turtles, as well as the human populations that survived and developed during those periods.

## Supplemental Information

10.7717/peerj.6123/supp-1 Supplemental Information 1 Supplemental information 1 to 7.

## Additional Information and Declarations

### Data Availability

The following information was supplied regarding data availability: The raw data are provided in the Tables.
