# Supplementary material for: Remains of Leatherback turtles, Dermochelys coriacea, at Mid-Late Holocene archaeological sites in coastal Oman: clues of past worlds
Source: PeerJ. 2018 Dec 17;6:e6123. doi: 10.7717/peerj.6123 (PMC6301280; doi:10.7717/peerj.6123)
Supplement: Supplemental Information 1 [file peerj-06-6123-s001.docx]

**Remains of Leatherback turtles, *Dermochelys coriacea*, at Mid-Late Holocene archaeological sites in coastal Oman: clues of past worlds**

**SUPPLEMENTAL INFORMATION**

**Supplemental Information 1**

**Brief taxonomy of the family Dermochelyidae**.

According to the Paleobiology Database page on *Dermochelyidae* (https://paleobiodb.org/classic/classify?taxon_no=37693), the presently recognized genera of the family, with respective numbers of species, are: *Arabemys* = 1 sp.; *Cosmochelys* = 1 sp.; *Egyptemys* = 2 spp.; *Eosphargis* = 3 spp.; *Maorichelys* = 1 sp.; *Mesodermochelys* = 1 sp.; *Natemys* = 1 sp.; *Psephorhorus* = 6 spp.; and *Turgaiscapha* = 1 sp. , see also Wood et al., (1996) and Eckert et al. (2012: 4-5).

**Supplemental Information 2**

**Re-evaluation of two Plio-Pleistocene fossils in the US National Museum, the only reported fossils of *Dermochelys*.**

The only fossils known to have been identified as “*Dermochelys* sp.” are two isolated ossicles from the Pliocene to Early Pleistocene site at Lee Creek Mine, North Carolina, USA, first described, and illustrated, in the unpublished PhD thesis of Köhler (1996: 130, 132, 151). These specimens are held in the United States National Museum (USNM), numbers USNM 358306 and USNM 358308. They were later mentioned in two paleontological publications as “*Dermochelys*” by Karl, Gröning & Brauchmann, (2012a: 166) and Karl, Lindow & Tütken (2012b: 214); however, these published and unpublished reports seem to have been missed by other workers.

At the request of JF, G. Zug & D. Bohaska, from the USNM re-examined the two ossicles reported as *Dermochelys* sp. by Köhler (1996),

They determined that one specimen [USNM 358306] is a fragment and considered to be identifiable only to the order Testudines; and the second [USNM 358308] is probably, but not unquestionably, from *Dermochelys* (Zug & Bohaska, *in litt*. to JF 18 June 2015).

Other evaluations, based on photos of these specimens (kindly provided by D. Bohaska), include three additional expert opinions:

R. Hirayama (*in litt.* to JF 24 September 2018) observed that the dorso-ventral thickness of the two ossicles in question appeared to be “between typical *Psephophorus* and extant *Dermochelys*”; he suggested that these specimens could be from a new species of *Dermochelys.*

R. Weems (*in litt.* to JF 24 September 2018) considered USNM 358308 to be consistent with *Dermochelys*, but he was not convinced that the characters seen on USNM 358306 are fully consistent with that taxon, and he suggested that the ossicles might be from a new species of *Dermochelys*.

J. Sterli (*in litt.* to JF 26 September 2018), using especially the comparative descriptions of ossicles in Delfino et al. (2013), and considering mainly the dorso-ventral thickness, suggested that the specimens in question are not *Dermochelys*.

In this light it is worth mentioning that there are reports of much older specimens that are undetermined, and in one case regarded to be between *Dermochelys* and “*Psephophorus*” (commonly considered to be a “wastebasket” taxon)*.* Barnes, Raschke & McLeod (1985) reported on Miocene marine vertebrate fossils from the Monterey Formation of the Los Angeles Basin. Included in their list of taxa form the vertebrate assemblage (pg. 17) is “aff. *Dermochelys* sp.”. They subsequently (pg. 18) explained that the “giant leatherback turtle fossils [are]closer to the modern genus *Dermochelys* than to the typical middle Miocene genus *Psephophorus*.” No further information seems to be available, although these specimens are apparently being studied (Velez-Juarbe, *in litt.* to JF 21 September 2018).

Wood et al. (2009) reported two even older specimens from South Carolina, USA: one from the Ashley Formation (Late Early Oligocene), and one from the Chandler Bridge Formation (Early Late Oligocene). In regard to the second, they stated: “this remarkable specimen clearly resembles the shell of the living *Dermochelys coriacea* far more closely than any other fossil dermochelyids that have so far been described.” No further details are available on these specimens, but there is an intention to begin working them up “in relatively short order” (Knight, *in litt.* to JF 1 October 2018).

In conclusion, there appear to be no known fossils of *Dermochelys coriacea*, although there are several fossils that have been described as “*Dermochelys* sp.”; “aff. *Dermochelys* sp.”; closer to *Dermochelys* than to *Psephophorus*; more like *Dermochelys coriacea* than any other fossil dermochelyids that have been described; and possibly a new species of *Dermochelys.* Once these undetermined specimen descriptions and identifications have been carefully worked out, the enigma of the missing fossil record of *D. coriacea* should be better understood.

**Supplemental Information 3**

**Clarification of enumerated archaeological sites at Ra’s al-Hamra.**

In 1973 a total of 12 archaeological sites were enumerated at Ra’s al-Hamra (Fig. 2). Nonetheless, site RH-9, was later found to be modern and not ancient (Biagi et al., 1984: 45; Uerpmann & Uerpmann, 2003: 8 fn 1; Munoz, 2014: 139 Table 4.3).

**Supplemental Information 4**

**Details of the archaeological contexts of the ossicles of *Dermochelys coriacea* found in Oman.**

In the case of RH-6, all 184 ossicles were at least 600 m from the present-day shore, and at least 7 m above present-day highest tide level; they were not found on a beach, but were considerable horizontal and vertical distance from the present-day beach, where they were recovered from the midst of a Neolithic site. Moreover, the 71 ossicles from Sector A (Table 1: collections 1 – 5) were only a few cm above the bedrock, more than 50 cm below the present surface, and associated with anthropogenic deposits and structural features; these were found densely concentrated, close to the remains of two dwellings. Most of the 72 ossicles from Sector C, recovered almost 1 m below the present surface, were associated with various cultural artefacts as well as traces of food-processing (Table 1: collections 10 & 13), especially over “living” floors (see Dibble et al. 1997), some of which were comprised of shells, including small gastropods (Table 1: collections 6, 7 & 15). Other ossicles from Sector C were recovered from a large pit, evidently used for refuse (Table 1: collection 8). Trench North provided 25 ossicles, which were found in compact deposits of fish bones and/or molluscs (Table 1: collection 17), on tabular gravelly floor-like surfaces comprised of compacted beach gravels (Table 1: collections19 & 22), and in proximity of structural remains (Table 1: collections 18, 20 & 21). The 25 ossicles from Trench North were all recovered at least 1.5 m below the present surface. In contrast to the 168 ossicles from Sectors A and C and Trench North, the 16 ossicles from Grave 7, in Sector B (Table 1: collection 24), are not unambiguously attributed to anthropogenic activities, other than being included in material used to fill the cenotaph.

The 30 ossicles from Sector C that were recovered from occupational floors, and 14 ossicles from Trench North retrieved from gravelly floor-like surfaces, warrant further comment. Most of the beach-derived materials that comprised these floors (e.g., mollusc shells and beach derived gravels) were much smaller than the smallest *D. coriacea* ossicles. Hence, aeolian transport of ossicles onto these floors would have been highly unlikely. Moreover, none of the 184 ossicles from RH-6 showed significant signs of surface abrasion, such as would have been produced by aeolian, or tidal, transport across a beach, to be deposited inland into the archaeological site. Similarly, none of these ossicles showed signs of tooth marks, which would have been made by scavengers that might have transported ossicles inland from a cadaver on the beach to the archaeological site. As mentioned above, in most cases the RH-6 ossicles were found in close physical association with diverse objects and structures that are clearly cultural artefacts and/or ecofacts. Hence, although the non-anthropogenic occurrence of some *D*. *coriacea* ossicles at certain loci at RH-6 cannot be ruled out, the vast majority of ossicles recovered from this site – over 90% – were clearly associated with a variety of archaeological contexts, having been found together with a wide variety of anthropogenic objects and structures, such as shell and stone beads, bone and stone tools, shell fish hooks, shell scrapers, gorge hooks, and other types of worked shells, as well as on occupational floors. In summary, the preponderance of ossicles from RH-6 are not simply explained as incidental components of beach debris, but rather as anthropogenic objects, related to the human activities at RH-6.

In regard to the 149 ossicles recovered from Ra’s al-Hadd, there was even more compelling evidence of anthropogenic origin for the remains. All these ossicles were at least 200 m from the present shoreline, and at least 90% of them were recovered from deposits clearly associated with past human occupational activities. For example, Room 33, a restricted indoor space 2.3 x 1.5 m (Fig. 4), yielded not only dense concentrations of shell and stone jewellery materials, but also 94 ossicles, (representing nearly two thirds of all ossicles from Ra’s al-Hadd), and these included 14 ossicles that were still articulated when discovered (Table 2: collection 1; Fig. 6). Two other ossicles came from a slightly larger closed indoor space, Room 54 (Table 2: collection 6). Both these spaces, which were in use at the time their respective deposits were made, were enclosed by solid walls, as they were part of permanent buildings, constructed with adobe. Thus, it would have been nearly impossible for a marine turtle to enter these restricted spaces; and the chances of beach debris being casually deposited therein would also have been minute; to begin with, aeolian processes would be unlikely to transport objects as large as ossicles the distance required to move from the beach to inside the buildings. Also, there were no conspicuous signs of surface abrasion on any of the HD-6 ossicles, which would have been produced by aeolian, or tidal, transport along a beach, and then conveyed inland into HD-6 buildings. Moreover, both of these rooms present clayey floors that had sealed the evidence above and below the respective floor; an accumulation of beech debris (including ossicles) would be highly unlikely while these rooms were occupied. A second large aggregate, totalling 35 ossicles, was recovered from F/G Courtyard (Table 2: collections 2 & 3), a partially closed space, a little more than twice the size of Room 33, where they were in close physical association with anthropogenic plant and animal remains. Three additional ossicles were also recovered from the partially closed F/G Courtyard, but from a later stratum (Table 2: collection 7). A total of nine ossicles was recovered from outside of building remains (i.e., not in closed spaces), most of which were from mixed sediments, thought to be from between known occupational phases (Table 2: collections 4, 5 & 8). Finally, six ossicles were retrieved from T.T.2/97 (Table 2: collection 9), a much later, open occupational space.

In summary, 134 ossicles (90% of the total from HD-6) were recovered from closed spaces (Room 33, Room 54, and F/Y Courtyard), within sealed contexts, and often in close association with cultural and faunal artefacts, so clearly of anthropogenic origin. The remaining 15 ossicles from HD-6 were found outside buildings and in Test Trench 2/97, so these were less obviously of archaeological significance. Nonetheless, it is highly unlikely that nesting *Dermochelys coriacea* (only nesting *D. coriacea* crawl out onto a beach) would have crawled all the way up the beach and into the Bronze Age settlement. Although it is possible (even if unlikely) that disarticulated ossicles from stranded, decomposed turtles could have been introduced into open settlement areas by scavenging animals, none of the 149 ossicles from HD-6, showed signs of tooth marks, which would have been left by scavengers that might have carried ossicles from the beach to inside the HD-6 site. In conclusion, there is no evidence that ossicles from HD-6 were derived from beach debris; rather all evidence confirms archaeological contexts of these small bony remains.

**Supplemental Information 5**

**Summary of the “maximum distinction method” (Grayson, 1973: 433) used to estimate the minimum number of individual (MNI) *Dermochelys coriacea* documented at RH-6 and HD-5, Oman.**

The dermal ossicles that form the epithecal mosaic of the carapace (dorsal shell), and to a lesser extent the plastron (ventral shell), of *D. coriacea* (Figs. 6 & 7) are distinctive at the species level, but it is not possible to determine if individual ossicles derive from an individual turtle. Furthermore, there is no known method to determine sex from ossicles; and although age class differences could possibly be determined, this would require detailed microstructural analysis (Delfino et al., 2013: 776). Hence, with the present state of knowledge the usual types of morphological comparisons that are used by zooarchaeologists to estimate the minimum number of individuals (MNI) of a particular species (Grayson, 1979: 205 ff) cannot be applied to ossicles of *D. coriacea*. Moreover, in theory, a single individual of *D. coriacea* could have provided all the ossicles that were recovered in the present study – those from RH-6 together with those from HD-6; there are estimated to be well over 5,000 ossicles in a single turtle (JF pers. obs; see Fig. 7A).

In this light, another approach is warranted: the definition of spatial-temporal boundaries to distinguish certain groups (or aggregates) of ossicles from other groups as having come from different individual turtles; or as Grayson (1973: 433) described: using a “maximum distinction method” to estimate the minimum number of individuals recovered from an archaeological study. For example, the specimens recovered from Sector A of RH-6 (Table 1, collections 1-5), the oldest context in which ossicles were recorded in this study, are distinguishable from the collection at T.T.2/97 of HD-6 (Table 2, collection 9), the youngest context in which ossicles were recorded in this study. These two groups of ossicles are separated by about 3 millennia and also 300 km.

The procedure can be further refined so that different collections (“aggregates”) of ossicles from the same site can be distinguished as having derived from distinct individuals. For example, collections from different periods at RH-6 can be distinguished, resulting in the conclusion that no less than six individual *D. coriacea* had contributed to the total of 184 ossicles recovered from the site, deposited during a nearly 2000-year span, from approximately 5600 to 3800 BC; that is: collections 1–5 from Period A-II; collections 6–13 from Period C-V; collections 14–16 from Period C-VI; collections 17–21 from Period TN-1; collection 22 from Period TN-II; and collection 23 from Period TN-IV (see Table 1). Similarly, distinguishing ossicles from different subphases at HD-6 would mean that no less than four, or five, individual *D. coriacea* had contributed to the total of 149 ossicles, deposited during about a 500-year period, from approximately 3000 to 2500 BC; that is: collection 1 from Subphase I-3b; collections 2–4 from Subphase I-3c; collections 5–7 from Subphase I-4a; collection 8 from Subphase I-4b; and collection 9 from Phase II (see Table 2). If even more refined criteria were used for establishing spatial-temporal boundaries, taking into account, for instance, the spatial locations of the ossicles within the site, then one could argue that the remains of somewhere between six and 24 individual *D. coriacea* had been recovered from RH-6, and the remains of between four and nine individual turtles were retrieved from HD-6.

However, several caveats must be considered when interpreting these numbers. As Grayson (1973: 438) explained, the maximum distinction method is subject to overestimation in that arbitrary excavation units are assumed to be independent, when in fact the remains of a single individual could potentially be recovered from more than one excavation unit. In the present case, this error was highly unlikely (see below).

Another concern is the perturbation of strata – either from anthropogenic or other actions – that could result in the introduction of bones from older deposits into younger deposits, or vice versa; hence, the ossicles from a single turtle could potentially be retrieved from two or more different stratigraphic units. In the case of HD-6, the ossicle-bearing contexts were spatially separated by at least 15–20 m (Fig. 4); and it is highly unlikely that mixing occurred between these areas, unless it was the result of human action – for which there is no evidence. For example, T.T.2/1997 is located beyond the ancient stone wall bordering the site to the north; the areas reported as “Outside of buildings” lay about 15 m from the other contexts considered here; Room 33 is part of a building located in the south-eastern area of the settlement, while Room 54 lies in the south-western sector. In addition, as reported above, both Room 33 and Room 54 were closed contexts (Fig. 4), revealing several clayey floors that have segregated the evidence both above and below the respective floor. The situation is somewhat different for F/G courtyard, which has yielded two collections attributed to two distinct phases; nonetheless, the deposits that yielded ossicles are separated by clayey floors as well as several stratigraphic units in between; hence, it is unlikely that stratigraphic mixing had any major effect, so either one or two individual turtles could have provided the total of four ossicles from F/G courtyard. Moreover, the temporal spans between each of the main ossicle-bearing occupations (i.e., between Phase I-3 and Phase I-4, as well as between either Phase I-3 or Phase I-4 and Period II), are too large for deliberate displacement to have occurred from one context to another. Although there is no evidence that this sort of stratigraphic mixing of artefacts or ecofacts at either RH-6 or HD-6, this possible complication in the interpretation of distinctions between aggregates based on stratigraphic data must be born in mind.

Finally, numbers used for estimating the minimum number of individuals (MNI) – however they may be obtained – represent the minimum number of individuals recovered from the archaeological sampling under study. They cannot pretend to estimate the number of individuals deposited at the site.

In summary, it is reasonable to conclude that remains of no less than six – possibly as many as 24 – individual *D. coriacea* were recovered at RH-6, and remains of no less than four or five – possibly as many as nine – of these turtles were retrieved from HD-6, all derived from the period of approximately 5600 to 2600 BC. This would translate to a “rough average minimum deposition rate” (RAMDR) of no less than one *D. coriacea* every 300 years, and as many as one every 75 years at RH-6, and no less than one *D. coriacea* every 125 years, and up to as many as one every 55 years at HD-6. Hence, although the absolute number of ossicles is less for HD-6 than for RH-6, *D. coriacea* remains were evidently deposited more rapidly at HD-6. However, such comparisons of deposition rates between sites is based on the assumption that volumes of substrate sampled at the sites under study are comparable, but this information is not available in this case.

References

**Barnes LG, Raschke RE, McLeod SA. 1985.** A Late Miocene marine vertebrate assemblage from Southern California. *National Geographic Society Research Reports* **21**: 13-20.**Wood RC, Johnson-Grove J, Gaffney ES, Maley KF. 1996.** Evolution and phylogeny of leatherback turtles (Dermochelyidae), with descriptions of new fossil taxa. *Chelonian Conservation and Biology* **2/2:** 266­286.

**Biagi P, Torke W, Tosi M, Uerpmann HP. 1984.** Qurum: A case study of coastal archaeology in Northern Oman. *World Archaeology* **16/1:** 43-61. DOI: 10.1080/00438243.1984.9979915.

**Delfino M, Scheyer TM, Chesi F, Fletcher T, Gemel R, MacDonald S, Rabi M, Salisbury SW. 2013.** Gross morphology and microstructure of type locality ossicles of *Psephophorus polygonus* Meyer, 1847 (Testudines, Dermochelyidae). *Geological Magazine* **150/5:** 767-782. DOI: 10.1017/S001675681200091X.

**Dibble HL, Chase PG, McPherron SP, Tuffreau A.** **1997**. Testing the reality of a “Living Floor” with archaeological data. *American Antiquity* **62/4**: 629-651. DOI: 10.2307/281882

**Eckert KL, Wallace BP, Frazier JG, Eckert SA, Pritchard PCH. 2012.** *Synopsis of the biological data on the leatherback sea turtle (Dermochelys coriacea)*. Washington DC: U.S. Department of Interior, Fish and Wildlife Service; xii, 158 pp.

**Grayson DK. 1973.** On the methodology of faunal analysis. *American Antiquity* **38/4:** 432-439. DOI: 10.2307/279149.

**Grayson DK. 1979.** On the quantification of vertebrate archaeofaunas. *Advances in Archaeological Method and Theory* **2:** 199-237.

**Karl H-V, Gröning E, Brauckmann C. 2012a.** New materials of the giant sea turtle *Allopleuron* (Testudines: Chelonioidea from the marine Late Cretaceous of Central Europe and the Palaeogene of Kazakhstan. *Studia Palaeocheloniologica* **4:** 153-173.

**Karl H-V, Lindow BE, Tütken T. 2012b.** Miocene leatherback turtle material of the genus Psephophorus (Testudines: Dermochelyoidea) from the Gram Formation (Denmark). *Studia Palaeocheloniologica* **4:** 205-216.

**Köhler R. 1996.** Eocene whales and turtles from New Zealand, pp. xv, 359. PhD. Thesis, Department of Geology, University of Otago, Dunedin, New Zealand. http://theses.otagogeology.org.nz/items/show/324

**Munoz O. 2014.** *Pratiques funéraires et paramètres biologiques dans la Péninsule d’Oman du Néolithique à la fin de l’âge du Bronze ancien (V-IIIe millénaires av. n.e.).* D. Phil. Thesis, University of Paris 1 Panthéon-Sorbonne; **Vol 1: 522 pp, Vol 2: 333 pp**, abstract available at: https://www.academia.edu/5944507/Pratiques_fun%C3%A9raires_et_param%C3%A8tres_biologiques_dans_la_p%C3%A9ninsule_d_Oman_du_N%C3%A9olithique_%C3%A0_la_fin_de_l_%C3%A2ge_du_Bronze_ancien_Ve-IIIe_mill%C3%A9naires_av._N.E._.

**Uerpmann HP, Uerpmann M. 2003.** *Stone Age Sites and their Natural Environment. The Capital Area of Northern Oman, Part III*. Wiesbaden: Ludwig Reichert; xiii, 265 pp.

**Uerpmann M, Uerpmann HP. 2007.** Shell midden economy in the 4th millennium BC. In: Cleuziou S, Tosi M, eds. *In the Shadow of the Ancestors. The Prehistoric Foundations of the Early Arabian Civilization in Oman.* Muscat: Ministry of Heritage and Culture, 103-104.

**Wood RC, Johnson-Grove J, Gaffney ES, Maley KF. 1996.** Evolution and phylogeny of leatherback turtles (Dermochelyidae), with descriptions of new fossil taxa. *Chelonian Conservation and Biology* **2/2:** 266­-286

**Wood RC, Knight JL, Cicimuri D, Sanders A. 2009.** Fossil leatherback turtles (Dermochelyidae) from the Paleogene of South Carolina. In: Braman DR (compiler) *Turtle Symposium, October 17-18, 2009. Abstracts and Program.* Special Publication of the Royal Tyrrell Museum. Drumheller, Alberta, Canada, 195-198.
